# Author Correction: dsRNAi-mediated silencing of *PIAS2beta* specifically kills anaplastic carcinomas by mitotic catastrophe

**DOI:** 10.1038/s41467-024-50135-0

**Published:** 2024-07-11

**Authors:** Joana S. Rodrigues, Miguel Chenlo, Susana B. Bravo, Sihara Perez-Romero, Maria Suarez-Fariña, Tomas Sobrino, Rebeca Sanz-Pamplona, Román González-Prieto, Manuel Narciso Blanco Freire, Ruben Nogueiras, Miguel López, Laura Fugazzola, José Manuel Cameselle-Teijeiro, Clara V. Alvarez

**Affiliations:** 1 Neoplasia & Endocrine Differentiation, Centro de Investigación en Medicina Molecular y Enfermedades Crónicas (CIMUS), University of Santiago de Compostela (USC), Instituto de Investigación Sanitaria (IDIS), Santiago de Compostela, Spain; 2 Department of Proteomics, Complejo Hospitalario Universitario de Santiago de Compostela (CHUS), Servicio Galego de Saúde (SERGAS), Instituto de Investigación Sanitaria de Santiago (IDIS), University of Santiago de Compostela (USC), Santiago de Compostela, Spain; 3 Department of NeuroAging Group – Clinical Neurosciences Research Laboratory (LINC), Complejo Hospitalario Universitario de Santiago de Compostela (CHUS), Servicio Galego de Saúde (SERGAS), Instituto de Investigación Sanitaria de Santiago (IDIS), University of Santiago de Compostela (USC), Santiago de Compostela, Spain; 4 Centro de Investigación Biomédica en Red en Enfermedades Neurodegenerativas, Instituto de Salud Carlos III, 28029 Madrid, Spain; 5 University Hospital Lozano Blesa, Institute for Health Research Aragon (IISA), ARAID Foundation, Aragon Government and CIBERESP, Zaragoza, Spain; 6 Centro de Investigación Biomédica en Red en Epidemiología y Salud Pública, Instituto de Salud Carlos III, 28029 Madrid, Spain; 7 Cell Dynamics and Signaling Department, Andalusian Center for Molecular Biology and Regenerative Medicine, Universidad de Sevilla - CSIC - Universidad Pablo de Olavide-Junta de Andalucía, 41092 Sevilla, Spain; 8 Department of Cell Biology, Faculty of Biology, University of Sevilla, 41012 Sevilla, Spain; 9 Department of Surgery, Complejo Hospitalario Universitario de Santiago de Compostela (CHUS), Servicio Galego de Saúde (SERGAS), Instituto de Investigación Sanitaria de Santiago (IDIS), University of Santiago de Compostela (USC), Santiago de Compostela, Spain; 10 Molecular Metabolism, Centro de Investigación en Medicina Molecular y Enfermedades Crónicas (CIMUS), University of Santiago de Compostela (USC), Instituto de Investigación Sanitaria (IDIS), Santiago de Compostela, Spain; 11 NeurObesity, Centro de Investigación en Medicina Molecular y Enfermedades Crónicas (CIMUS), University of Santiago de Compostela (USC), Instituto de Investigación Sanitaria (IDIS), Santiago de Compostela, Spain; 12 Department of Endocrine and Metabolic Diseases and Laboratory of Endocrine and Metabolic Research, Istituto Auxologico Italiano, Istituto Di Ricovero e Cura a Carattere Scientifico (IRCCS); Department of Pathophysiology and Transplantation, University of Milan, Milan, Italy; 13 Department of Pathology, Complejo Hospitalario Universitario de Santiago de Compostela (CHUS), Servicio Galego de Saúde (SERGAS), Instituto de Investigación Sanitaria de Santiago (IDIS), University of Santiago de Compostela (USC), Santiago de Compostela, Spain; 14 Present Address: Dana Farber Cancer Institute, Boston, MA USA

**Keywords:** Cancer therapy, Thyroid cancer, Mitosis

Correction to: *Nature Communications* 10.1038/s41467-024-47751-1, published online 14 May 2024

The original version of the Supplementary Information associated with this Article included an incorrect Supplementary Movie 1 file, in which the second part contained the heading “ATC cell line B-CPAP” instead of “PDTC cell line B-CPAP”. The HTML has been updated to include a corrected version of Supplementary Movie 1; the original incorrect version of Supplementary Movie 1 can be found as Supplementary Information associated with this Correction.

### Supplementary Information

Supplementary Movie 1

