# Supplementary material for: dsRNAi-mediated silencing of PIAS2beta specifically kills anaplastic carcinomas by mitotic catastrophe
Source: Nat Commun. 2024 May 14;15:3736. doi: 10.1038/s41467-024-47751-1 (PMC11094195; doi:10.1038/s41467-024-47751-1)
Supplement: Supplementary file 1 — Supplementary information [file 41467_2024_47751_MOESM1_ESM.pdf]

Supplementary Information

dsRNAi-mediated silencing of *PIAS2beta* specifically kills anaplastic carcinomas by mitotic catastrophe

Joana S Rodrigues[1 13 15], Miguel Chenlo[1 15], Susana B Bravo[2], Sihara Perez-Romero[1], Maria, Suarez-Fariña[1], Tomas Sobrino[3 4], Rebeca Sanz-Pamplona[5 14], Román González-Prieto[6 7], Manuel Narciso Blanco Freire[8], Ruben Nogueiras[9], Miguel López[10], Laura Fugazzola[11], José Manuel Cameselle-Teijeiro[12 16], Clara V Alvarez[1 16]

1 Neoplasia & Endocrine Differentiation, Centro de Investigación en Medicina Molecular y Enfermedades Crónicas (CIMUS), University of Santiago de Compostela (USC), Instituto de Investigación Sanitaria (IDIS), Santiago de Compostela, Spain

2 Department of Proteomics, Complejo Hospitalario Universitario de Santiago de Compostela (CHUS), Servicio Galego de Saúde (SERGAS), Instituto de Investigación Sanitaria de Santiago (IDIS), University of Santiago de Compostela (USC), Santiago de Compostela, Spain

3 Department of NeuroAging Group – Clinical Neurosciences Research Laboratory (LINC), Complejo Hospitalario Universitario de Santiago de Compostela (CHUS), Servicio Galego de Saúde (SERGAS), Instituto de Investigación Sanitaria de Santiago (IDIS), University of Santiago de Compostela (USC), Santiago de Compostela, Spain

4 Centro de Investigación Biomédica en Red en Enfermedades Neurodegenerativas, Instituto de Salud Carlos III, 28029 Madrid, Spain

5 University Hospital Lozano Blesa, Institute for Health Research Aragon (IISA), ARAID Foundation, Aragon Government, Zaragoza, Spain

6 Cell Dynamics and Signaling Department, Andalusian Center for Molecular Biology and Regenerative Medicine, Universidad de Sevilla - CSIC - Universidad Pablo de Olavide-Junta de Andalucía, 41092 Sevilla, Spain

7 Department of Cell Biology, Faculty of Biology, University of Sevilla, 41012 Sevilla, Spain

8 Department of Surgery, Complejo Hospitalario Universitario de Santiago de Compostela (CHUS), Servicio Galego de Saúde (SERGAS), Instituto de Investigación Sanitaria de Santiago (IDIS), University of Santiago de Compostela (USC), Santiago de Compostela, Spain

9 Molecular Metabolism, Centro de Investigación en Medicina Molecular y Enfermedades Crónicas (CIMUS), University of Santiago de Compostela (USC), Instituto de Investigación Sanitaria (IDIS), Santiago de Compostela, Spain

10 NeurObesity, Centro de Investigación en Medicina Molecular y Enfermedades Crónicas (CIMUS), University of Santiago de Compostela (USC), Instituto de Investigación Sanitaria (IDIS), Santiago de Compostela, Spain

11 Department of Endocrine and Metabolic Diseases and Laboratory of Endocrine and Metabolic Research, Istituto Auxologico Italiano, Istituto Di Ricovero e Cura a Carattere Scientifico (IRCCS); Department of Pathophysiology and Transplantation, University of Milan, Milan, Italy

12 Department of Pathology, Complejo Hospitalario Universitario de Santiago de Compostela (CHUS), Servicio Galego de Saúde (SERGAS), Instituto de Investigación Sanitaria de Santiago (IDIS), University of Santiago de Compostela (USC), Santiago de Compostela, Spain

13 Dana Farber Cancer Institute, Boston MA, USA

14 Centro de Investigación Biomédica en Red en Epidemiología y Salud Pública, Instituto de Salud Carlos III, 28029 Madrid, Spain

15 These authors contributed equally

16 These authors jointly supervised this work

Supplementary information index:

-Supplementary Figures 1-10

-Supplementary Figure 11-Graphical Abstract

-Unprocessed Scans of westerns from Supplementary Figures

Suppl Figure 1a – 1l

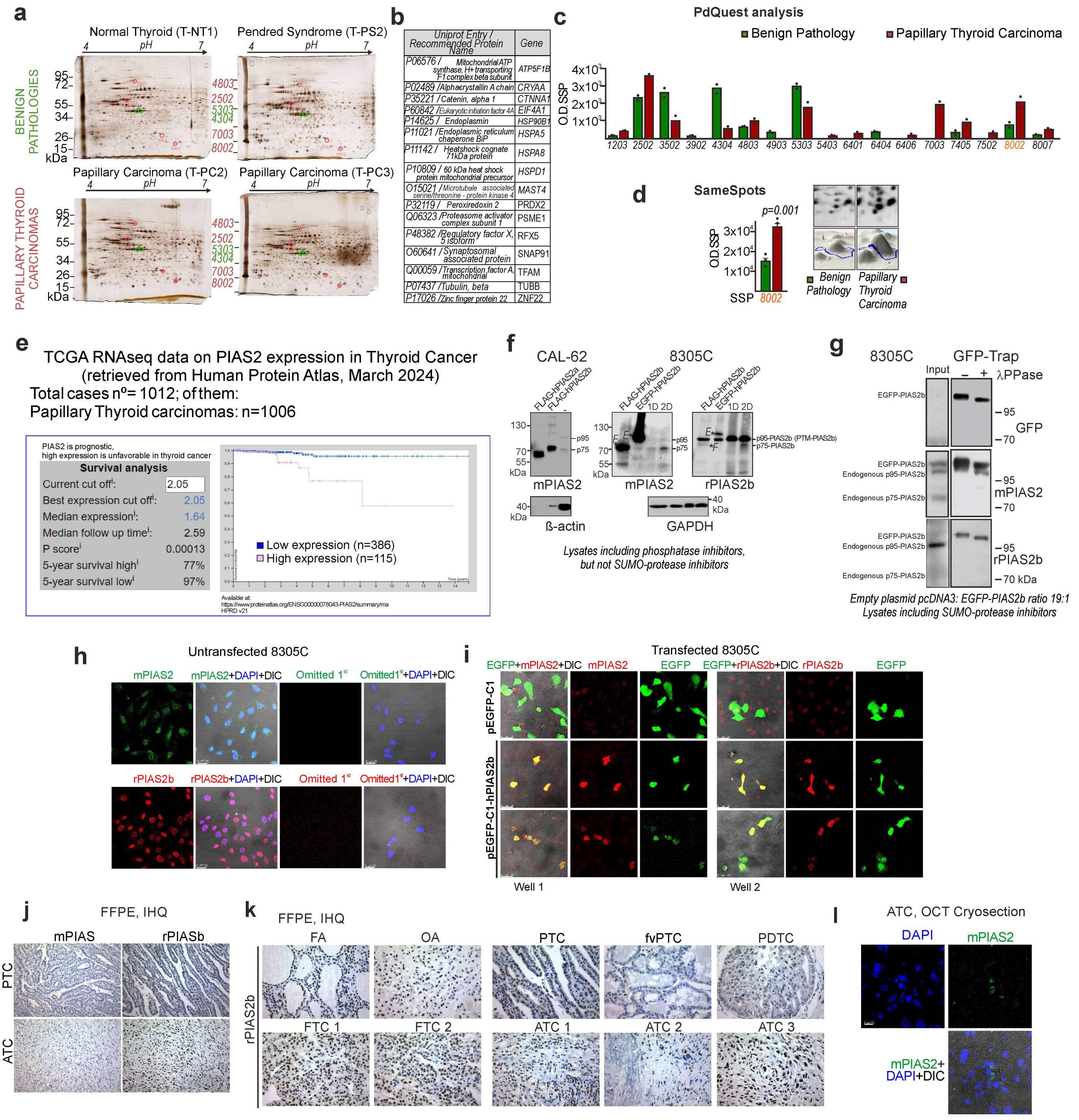

Supplementary Figure 1: The PIAS2b protein is differentially expressed in PTC. Validation of the two PIAS2b antibodies for western blot and staining. Selection of the most effective PIAS2b-dsRNAi anti-cancer conditions in commercially available thyroid cancer cell lines. Overexpression reveal PIAS2b as a dosage-sensitive protein in ATC cells. **(a)** Representative gels of the initial 2D proteomic study with primary cultures of normal (T-NT) / benign (T-PS) hyperplasia and differentiated PTC carcinomas (T-PC). Identified proteins increased in cancer are in red, and decreased in green. **(b)** Identified proteins unchanged in all cultures. **(c-d)** Spots (SSP) quantification with PDQuest and SameSpots. (In Table S2, the identification peptides after MALDI-TOF). The 8002 spot was PIAS2. **(e)** RNA-seq from the TCGA consortium study in thyroid carcinoma (retrieved from HPRD site at March 2024). High PIAS2 expression is an unfavourable prognostic factor in PTC. **(f)** Western blot in CAL-62 and 8305C ATC cell lines extracts (glycerol lysis buffer with phosphatase inhibitors) using small amounts of transfected FLAG-hPIAS2a (hPIAS2a Uniprot mass (Da. 63,396), FLAG-hPIAS2b (hPIAS2b Uniprot mass (Da. 68,240), or EGFP-hPIAS2b constructs as controls. The monoclonal mouse PIAS2 antibody (mPIAS2), with a common epitope in PIAS2a / PIAS2b, recognized a 75-kDa FLAG-hPIAS2b (F*), a <70-kDa FLAG-hPIAS2a, and the fusion EGFP-PIAS2b (E*). Despite using the same transfection amounts, FLAG-hPIAS2a expression was much higher than that of FLAG-hPIAS2b. mPIAS2 recognized two endogenous bands, p75- and p95- PIAS2b. rabbit PIAS2b antibody (rPIAS2b), raised against the serine-rich tail, strongly recognized the endogenous p95-PIAS2b protein, and with less intensity endogenous p75-PIAS2b or transfected FLAG-hPIAS2b, and EGFP-C1-hPIAS2b. In summary, the antibody mPIAS2 detects both endogenous p75-PIAS2b and post-translationally modified (PTM) p95-(PTM)-PIAS2b, and rPIAS2b, is better at detecting (PTM) p95--PIAS2b. PIAS2a was not detected. **(g)** GFP-Trap pull-down from transfected EGFP-PIAS2b (pcDNA3: pEGFP-C1-hPIAS2b, 19:1) lysed in a buffer with SUMO-protease inhibitors. Beads were divided into two halves and incubated with lambda phosphatase or buffer alone. GFP, mPIAS2 and rPIAS2b westerns were performed sequentially. The three antibodies recognized a single band after the Trap, that reduced weight after dephosphorylation. This indicates that PIAS2b is phosphorylated in cells but recognition was not affected by phosphorylation. In the Input, mPIAS2 was the most sensitive, recognizing Exogenous EGFP-PIAS2b and Endogenous p95-PIAS2b and p75-PIAS2b; rPIAS2b recognized best p95-PIAS2b, and less p75-PIAS2b or EGFP-PIAS2b; GFP antibody was not sensitive enough to detect small amount in the input. **(h)** Staining in asynchronous 8305C cells with mPIAS2 (green) and rPIAS2b (red). DAPI stains the nuclei; Differential interference Contrast (DIC). **(i)** Validation of mPIAS2 and rPIASb staining in cells transfected with pEGFP-C1 vector or with pEGFP-C1-hPIAS2b. EGFP signal is cell wide, not nuclear in pEGFP-C1. EGFP signal is nuclear in pEGFP-C1-hPIAS2b and colocalized with mPIAS2 or rPIAS2b. **(j)** Polyclonal rPIAS2, but not monoclonal mPIAS2, detects nuclear PIAS2b in FFPE tissue immunohistochemistry (IHQ). **(k)** The rPIAS2 antibody IHQ detected nuclear staining in all kind of thyroid tumors (ATC, anaplastic thyroid carcinoma; FA, follicular adenoma; FTC, follicular thyroid carcinoma; fvPTC, follicular variant of PTC; OA, oxyfilic adenoma; PDTC, poorly differentiated thyroid carcinoma; PTC, papillary thyroid carcinoma, classic variant). No quantitative differences were evaluated as some of the tissue-blocks were older. PDTC and ATC are rare cancers, and there are not enough recent samples for such a study. **(l)** The mPIAS antibody immunofluorescence, but not rPIAS2, stained mitotic cells in OCT cryosections of ATC.

Suppl Figure 1m – 1x

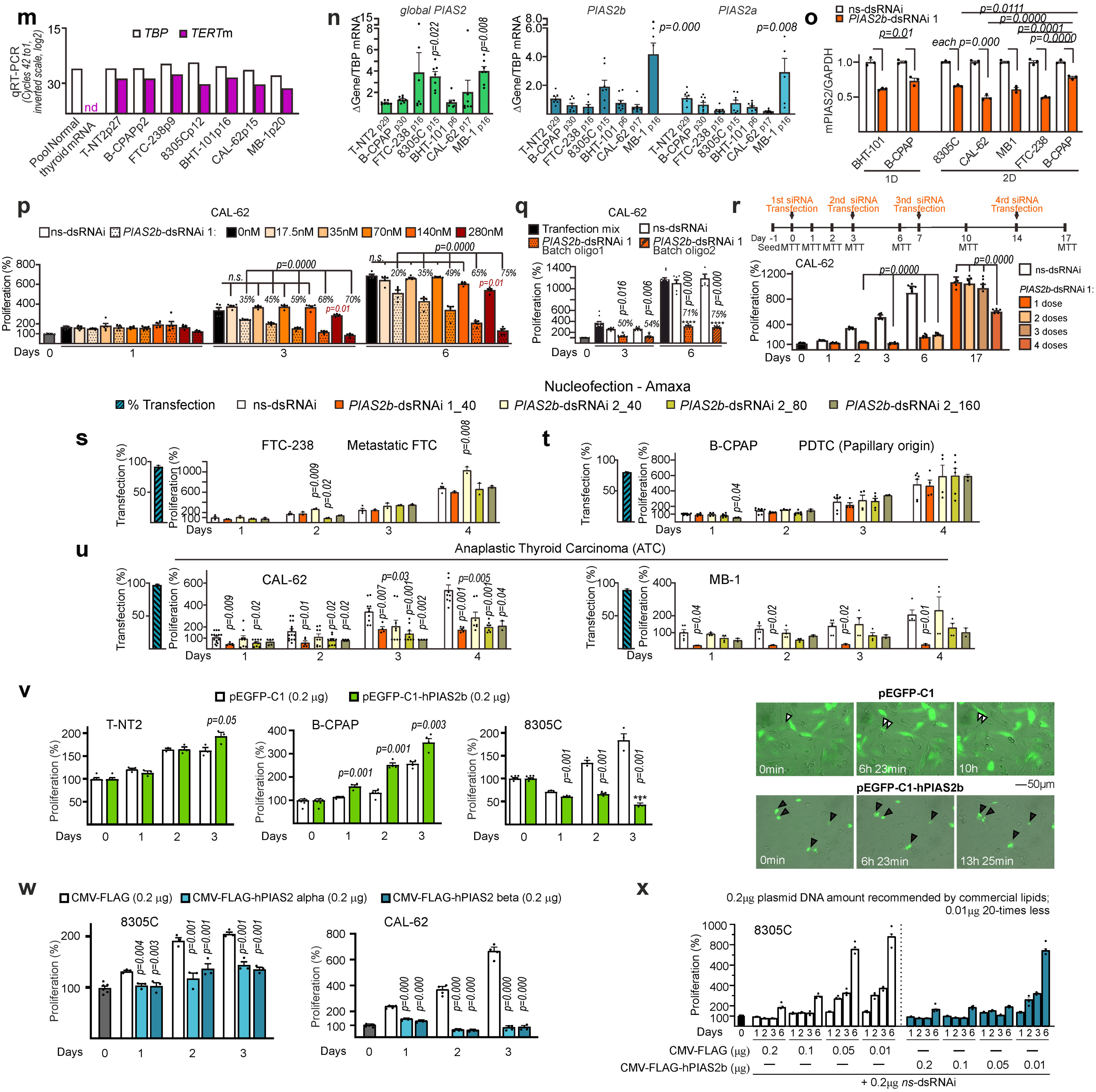

(m) *TERT* mRNA (*TERTm*) expression in the cell lines used in this study compared to T-NT2, a normal thyroid primary culture that spontaneously kept growing (and, correspondingly, express TERT). Expression was normalized by *TBP*, an homogeneous expressed gene. **(n)** Global *PIAS2* mRNA expression and its two isoforms in the cell lines compared to T-NT2. **(o)** Quantification of Western blots with a representative image in Figure 1h. BCPAP has significantly less PIAS2b reduction with *PIAS2b*-dsRNAi than any other cell line. **(p)** Dose-response cell growth study of the *PIAS2b*-dsRNAi 1. **(q)** Comparative cell growth study of different batches of *PIAS2b*-dsRNAi 1 prepared with two batchs of oligos from different commercial sources. **(r)** Time-course in vitro experiment where cells were transfected with one transfection, or with 2, 3 or 4 repeated doses once per week through 4 weeks. Although the efficiency of transfection diminishes due to cell accumulation in the well, the effect of PIAS2b-dsRNAi 1 is maintained. **(s–u)** Comparison of *PIAS2b*-dsRNAi 1 at half-dose to *PIAS2b*-dsRNAi 2 at increasing concentrations. Both sequences are effective only in ATC cell lines, and *PIAS2b*-dsRNAi 1 is the most effective. These experiments were performed with nucleofection. **(v-w)** PIAS2 is a dosage-sensitive protein in ATC. Transient transfection of pEGFP-C1-hPIAS2b **(v)** or Flag-hPIAS2 alpha or beta **(w)** induces cell death in 8305C and CAL-62 anaplastic thyroid cells but do not affect the normal thyroid T-NT2 cells or the poorly differentiated papillary thyroid carcinoma B-CPAP cell line. Transfection of the vector pEGFP-C1 or CMV-Flag does not affect cell growth in any of the cells. Right: Representative micrographs of time-lapse experiments demonstrate condensation and death of the EGFP-hPIAS2b 8305C anaplastic thyroid cancer transfected cells. **(x)** Dose-response cell growth study for PIAS2b dosage. Starting with the recommended 0.2 µg plasmid /well (indicated in the commercial lipid mix), progressive dilutions of pCMV-Flag or pCMV-Flag-hPIAS2b were compared in parallel, followed by the control ns-dsRNAi mix. Only 20-times less PIAS2b (0.01 µg plasmid /well) allowed cell growth.

For (c) n = 2, and (d) n = 3 patients' cultures per group; two-sided unpaired T-test. For (m) n = 1, (n), n = 7 and (o) n = 3 independent samples. For (p) n = 4, (q-r) n = 6, (s-t) n = 4, (u) n = 5 (CAL-62) or n = 4 (MB-1) independent experiments. For (v) n = 3 (T-NT2, 8305C) and n = 4 (BCPAP), for (w) n = 4, and (x) n = 3 independent experiments. (n) two-sided one-way ANOVA with Dunn's multiple correction; (o-p-q-r) two-sided one-way ANOVA with Tukey's multiple correction; (s-t-u) two-sided Mann-Whitney; (v) two-sided unpaired T-test; (w) two-sided one-way ANOVA with Dunnett's multiple correction. Bar indicate means (c) ± SEM (all other sections); exact p value is indicated in the figure. Source data are provided as a Source Data file.

Suppl Figure 2

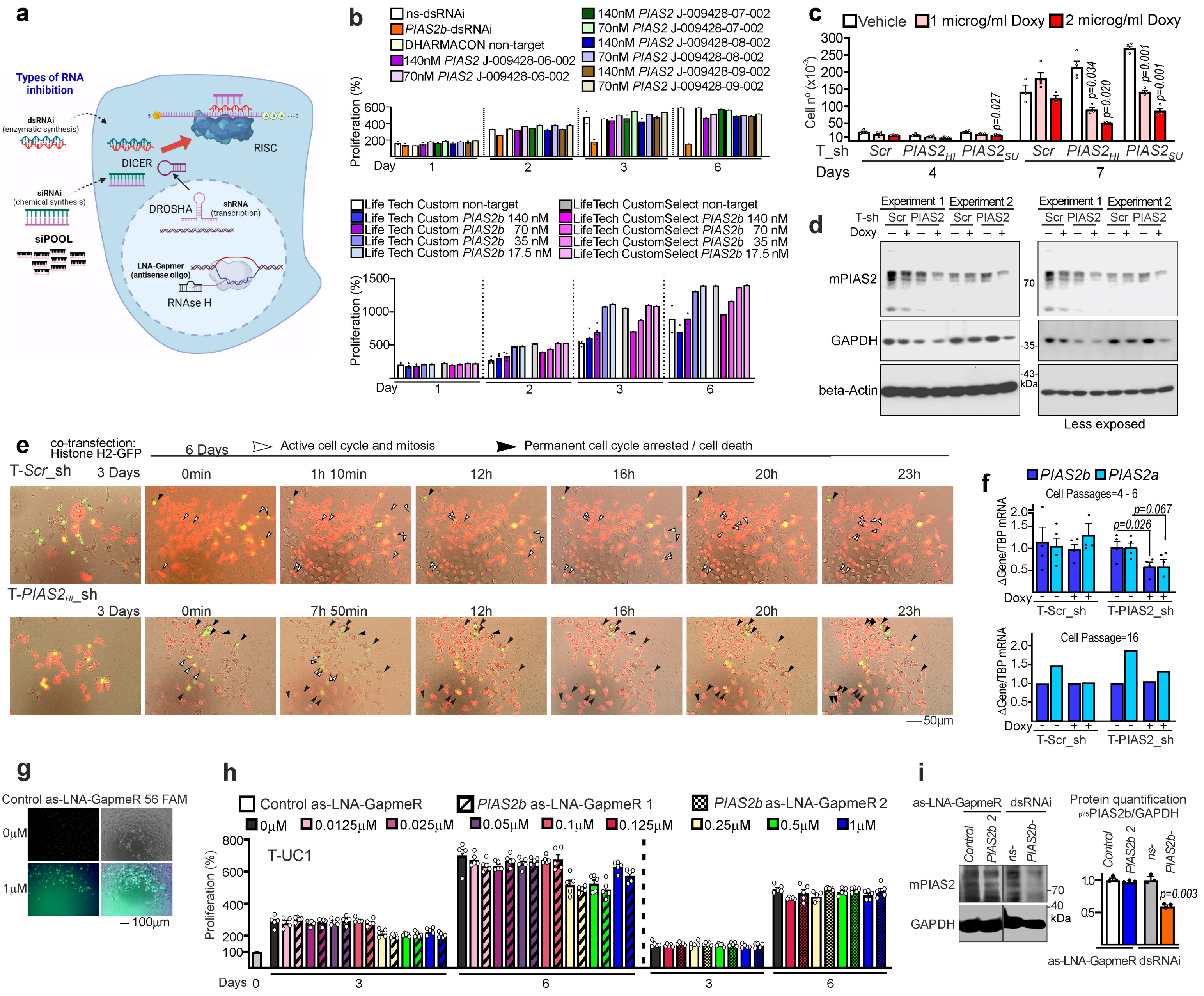

Supplementary Figure 2: Different strategies of PIAS2b downregulation. (a) Cartoon representing strategies to downregulate mRNA expression from intrinsic cellular pathways and synthetic strategies. Some of these components do not work properly in cancer cells. The target mRNA is transcribed by the RNA Pol II in a long chain including introns and exons; at this step, synthetic LNA-Gapmer antisense oligos (ASOs) bound to complementary chain induce RNA-H mediated degradation of the target RNA. The mature transcribed mRNA is exported to the cytoplasm. Again at the nucleus, microRNA genes or synthetic shRNA transfected genes are transcribed by RNA Pol III and processed by Drosha to become mature shRNAs exported to the cytoplasm. At the cytoplasm, shRNA are processed by DICER to become single-stranded short RNA, bound by the RISC complex to its target sequence in long mRNAs. This results in reduced translation, or degradation of the mRNA. Chemically synthesized double-stranded siRNA, or enzymatically transcribed double-stranded RNAi (dsRNAi) could be transfected into the cell cytoplasm, to be processed by DICER, selected as monocatenary interference RNA, attached to the RISC complex and bound to the target mRNA, similarly inducing its downregulation. siPOOLs are chemically synthesized 30 siRNA pools all targeted to different sequences of the same gene RNA but a very low concentration (total amount <5nm), with a strategy of higher efficacy, lower off-target effects. **(b)** Commercial siRNAs from chemical synthesis does not reduce *PIAS2b* nor affect 8305C proliferation. Top: Dharmacon On-Target set of 4 sequences PIAS2 siRNAs (J-009428) and non-target control compared to our dsRNAis. Bottom: Lif-technologies Custom prepared *PIAS2b* siRNAs using our sequence (Custom), and the same but with proprietary modified nucleotides giving enhanced half-life (Custom Select). **(c-f)** 8305C anaplastic cell populations expressing Doxycycline-inducible shRNA (TURBO) against a non-target control (Scr) or *PIAS2* were sorted by the intensity of co-expressed RFP marker in high (HI) or sufficient (SU) intensity. **(c)** One week after *PIAS2* shRNA induction a significant downregulation of cell growth was observed in both HI and SU populations, at two Doxycycline (Doxy) doses tested. **(d)** Western blot with mPIAS2 antibody showing reduction of PIAS2b protein after Doxycycline in the population expressing *PIAS2* shRNA but not in the non-target Scr shRNA controls. Shown are two independent experiments, and two film exposures. **(e)** Time-lapse experiments showed mitotic arrest and cell death in the Doxycycline-treated *PIAS2*-shRNA-HI population. Transfected Histone H2-GFP was used to better follow some mitoses. **(f)** Before passage 10, Doxycline reduced expression of both PIAS2 mRNA isoforms exclusively in cells expressing PIAS2 shRNA but not in the non-target Scr shRNA controls. This was lost at Passage 16. **(g-i)** *PIAS2b* as-LNA-GapmeR enter 8305C anaplastic cells but do not reduce cell number nor protein expression. **(g)** Control as-LNA-GameR oligo labeled with fluorescent 56-FAM showed passive concentration inside the cells. **(h)** Dose-response cell growth experiment using non-target Control as-LNA-Gapmer, and two oligos against *PIAS2b* (1, custom prepared at 3' UTR; 2, best sequence recommended by the company targeting all PIAS2 isoforms). **(i)** PIAS2b as-LNA-GameR2 is unable to reduce PIAS2b protein, while the in vitro transcribed *PIAS2b*-dsRNAi is effectively downregulating PIAS2b protein expression.

(a) own design from full license Biorenders.com templates. (b) Top, n=2; Bottom, n=3; (c- f- h) n = 4, independent experiments; (i) n = 3 samples. (c) and (h) Two-sided two-way ANOVA with Tukey's multiple comparison test; (f) and (i) wo-sided unpaired T-Test. Bar indicate means ± SEM; exact p value is indicated in the figure. Source data are provided as a Source Data file.

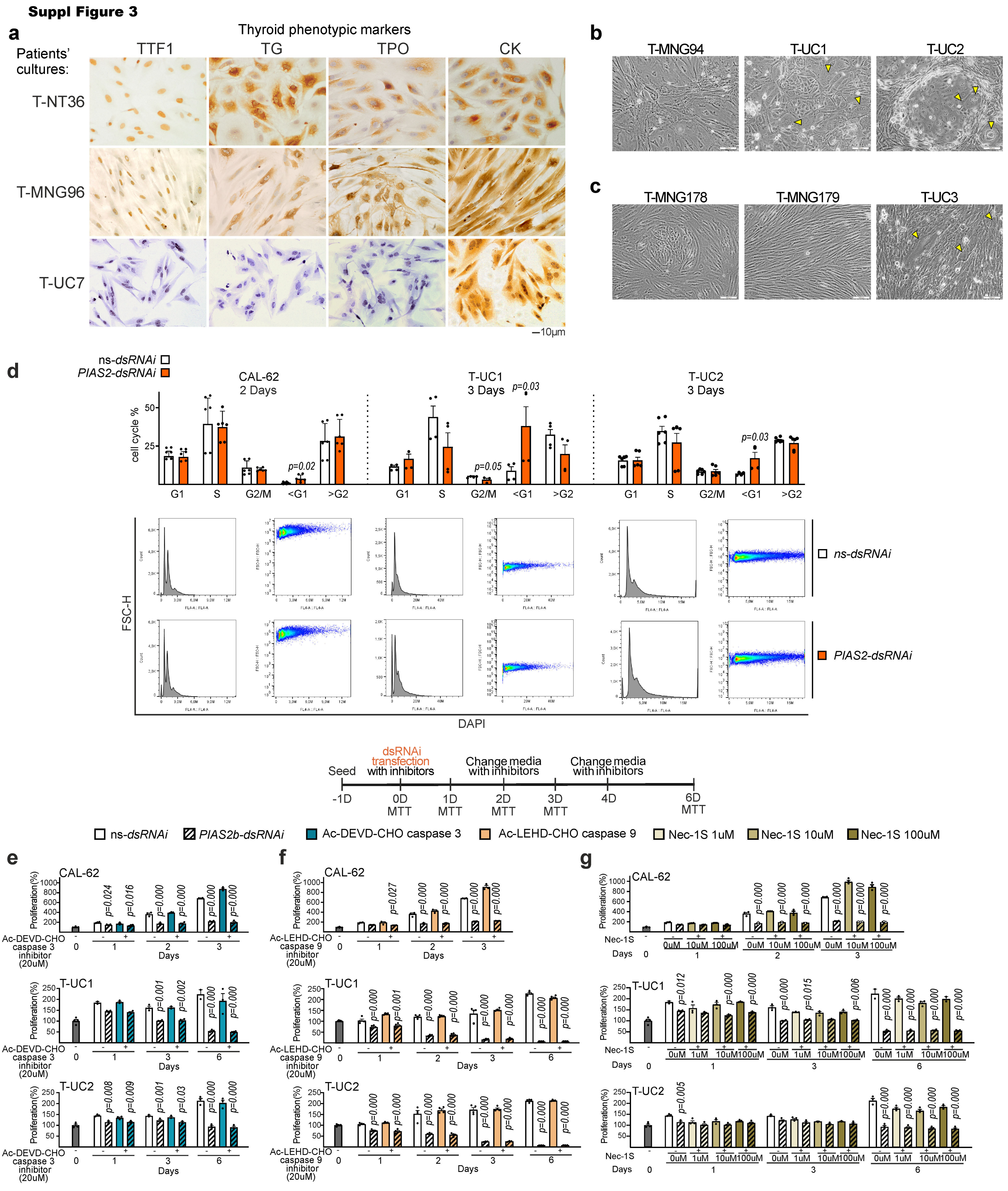

Supplementary Figure 3. The primary thyroid cultures grown in h7H conditions are > 95% follicular or cancer epithelium. *PIAS2b*-dsRNAi induces cell death not prevented by caspase or RIPK inhibitors. (a) Immunocytochemistry of primary cultures stained with thyroid-marker antibodies validated for clinical pathology, NKX2-1 (TTF1), thyroglobulin (TG), thyroperoxidase (TPO), and cytokeratins, AE1-AE3 (CK). Counterstaining with diluted hematoxylin. Shown is the comparison of a normal thyroid (T-NT36), a multinodular goiter (T-MNG96) and an ATC (T-UC7). All are epithelial cultures, but T-UC7 lacks markers of thyroid follicular differentiation. **(b-c)** The primary cultures grow accordingly to their pathological origin and maintain their original characteristics. **(b)** From Patient 1, T-MNG94, T-UC1 and T-UC2 were established. **(c)** From Patient 2, T-MNG178, T-MNG179 and T-UC3 were established. T-MNG cultures present elongated cells, well-ordered and organized in groups as follicular-like structures. T-UC cultures grow fast, with different populations, and giant cells in the middle (yellow arrows). **(d)** Flow cytometry analysis showed a significant increase in dead cells (<G1) after treatment with PIAS2b-dsRNAi (orange bars) compared to ns-dsRNAi (white bars). Shown are an ATC cell line (CAL-62) and two ATC primary cultures (T-UC1, T-UC2). **(e)** Caspase 3 (Ac-DEVD-CHO), **(f)** Caspase 9 (Ac-LEHD-CHO) or **(g)** RIPK (Nec-1S) inhibition does not block cell death induced by PIAS2b-dsRNAi at any dose tested.

(d) n = 6 (CAL-62, T-UC2) and n = 4 (T-UC1) independent experiments without replicates; (e and g) n = 3 , (f) n = 3 (CAL-62), n = 4 (T-UC1, T-UC2), independent experiments with multiple replicates. (d) Two-sided Mann-Whitney; (e-f-g) Two-sided two-way ANOVA with Tukey's multiple comparison test. Bar indicate means ± SEM; exact p value is indicated in the figure. Source data are provided as a Source Data file.

Suppl Figure 4

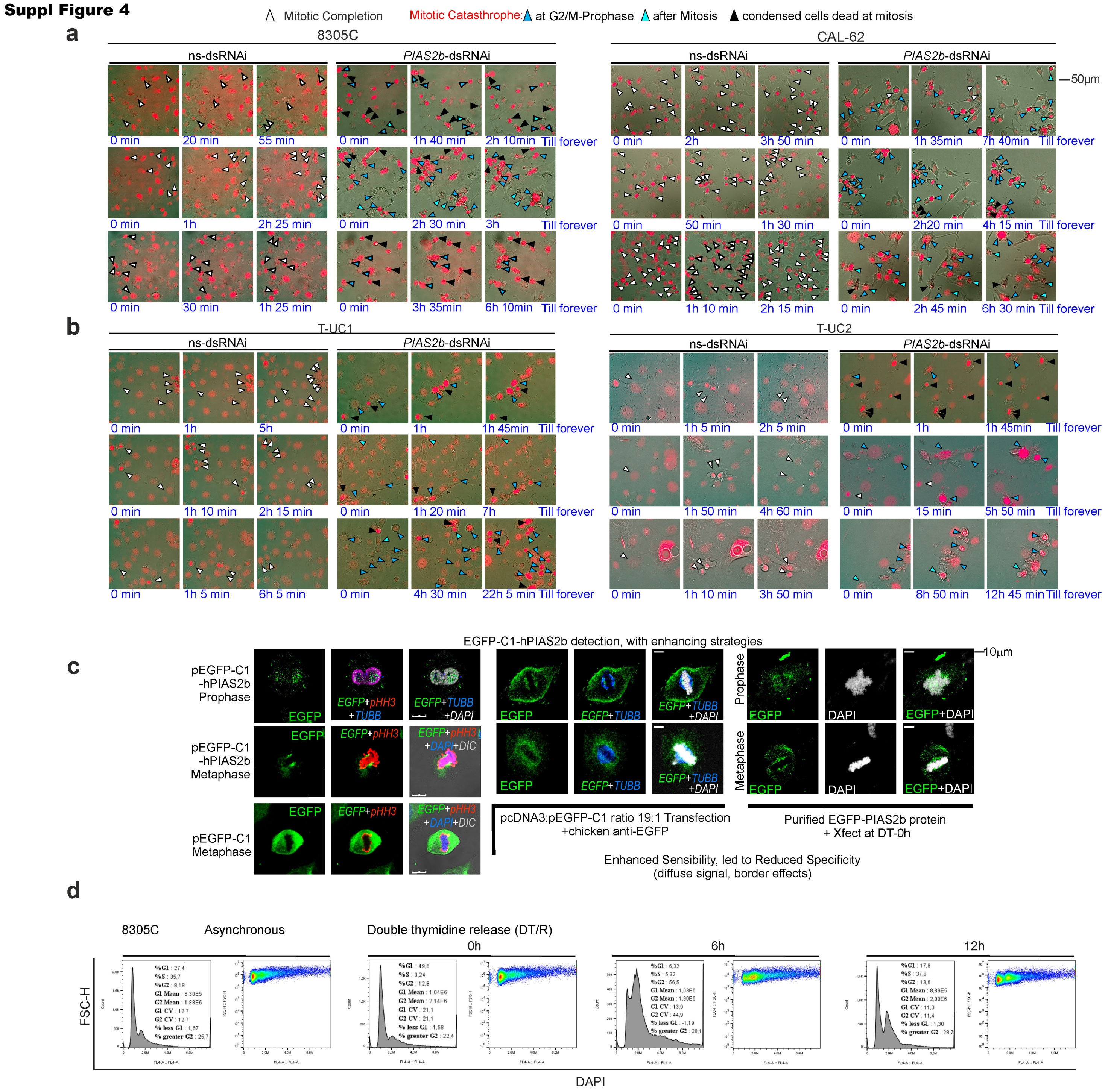

Supplementary Figure 4. Mitotic catastrophe induced by *PIAS2b*-dsRNAi. PIAS2b has a role in mitosis of human thyroid cells, both anaplastic cancer or normal thyroid. (a-b) Three fields from three different wells recorded by time-lapse in ns- and PIAS2b-dsRNAi treated ATC cells, cell lines **(a)** and patients' cultures **(b).** Selected fields and quantification are shown in main Figure 4. **(c)** Transfection of 8305C cells with pEGFP-C1-hPIAS2b at very low amounts to prevent cell death (Figure S1x) followed by enhancing signal strategies shown also in Figure 5; EGFP immunostaining, shows progressive EGFP localization at the spindle from Prophase to Metaphase compared to broad cytoplasmic staining in the metaphase of a EGFP-C1 transfected cell. Phospho-Histone H3 (pHH3) indicates mitotic cells. TUBB-647 (TUBB) is a directly labeled antibody detecting all Tubulin betas. Direct transfection with Xfect of GFP-Trap eluted EGFP-PIAS2b protein also detect progressive localization at the spindle. However, both enhancing strategies increased background with border effects and spotty or diffuse signal. **(d)** Flow cytometry validation of the double-thymidine block followed by shake-off and a release time-course (DT/R) (n = 4 independent experiments without replicates). 6 h after release (DT/R- 6 h) 56.5% of the 8305C cells were at G2/M. Source data are provided as a Source Data file.

Suppl Figure 5

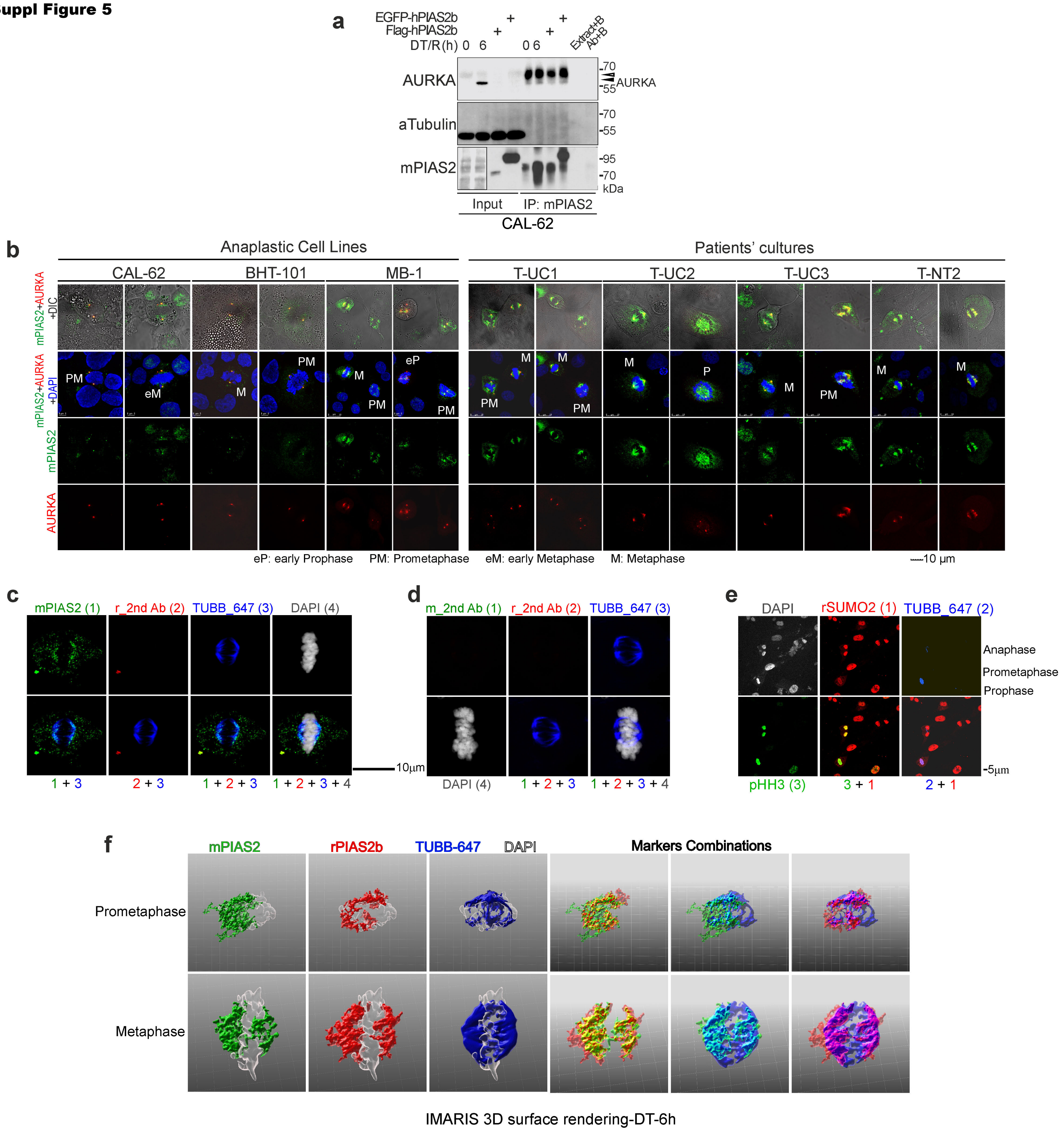

Supplementary Figure 5. PIAS2b at mitosis in anaplastic thyroid carcinoma: standardization of superresolution microscopy, and Imaris 3D volumetric rendering. (a) Pull-downs with mPIAS2 antibody in DT/R-6 h synchronized CAL-62 extracts indicate binding of AURKA and alpha Tubulin to PIAS2b at mitosis. AURKA and aTubulin was bound by transfected exogenous human PIAS2b detected with the mPIAS2 antibody. **(b)** PIAS2b and AURKA colocalization at mitosis of the three other human ATC cell lines: CAL-62, BHT-101 and MB-1, and patients' primary cultures from ATC (T-UC1, T-UC2, T-UC3) and normal thyroid (T-NT2) (n = 4 independent wells per culture; >100 cells per culture analysed). eP, early Prophase; PM, Prometaphase; eM, early Metaphase; M, Metaphase. **(c-d)** Negative controls for superresolution confocal microscopy showed in Figures 5 and 7. **(e)** SUMO2 (rSUMO2, red pseudocolor) colocalizes with cromatin as reveled by DAPI (grey pseudocolor) and phospho-Histone H3 (pHH3, green pseudocolor) colocalization, in the absence of spindle colocalization (TUBB_647, blue pseudocolor). **(f)** 3D volumetric rendering of two cells at mitosis, a Prometaphase and a Metaphase, stained with mPIAS2 and rPIAS2b, together with directly labeled TUBB-647 and DAPI. 3D surfaces of both antibodies were located at the centrosomal side of the spindle where maximal colocalization was achieved at Metaphase as shown by blended pseudocolors. Quantification on replicate cells during the progressive mitotic phases is shown in Figure 5. Source data are provided as a Source Data file.

Suppl Figure 6

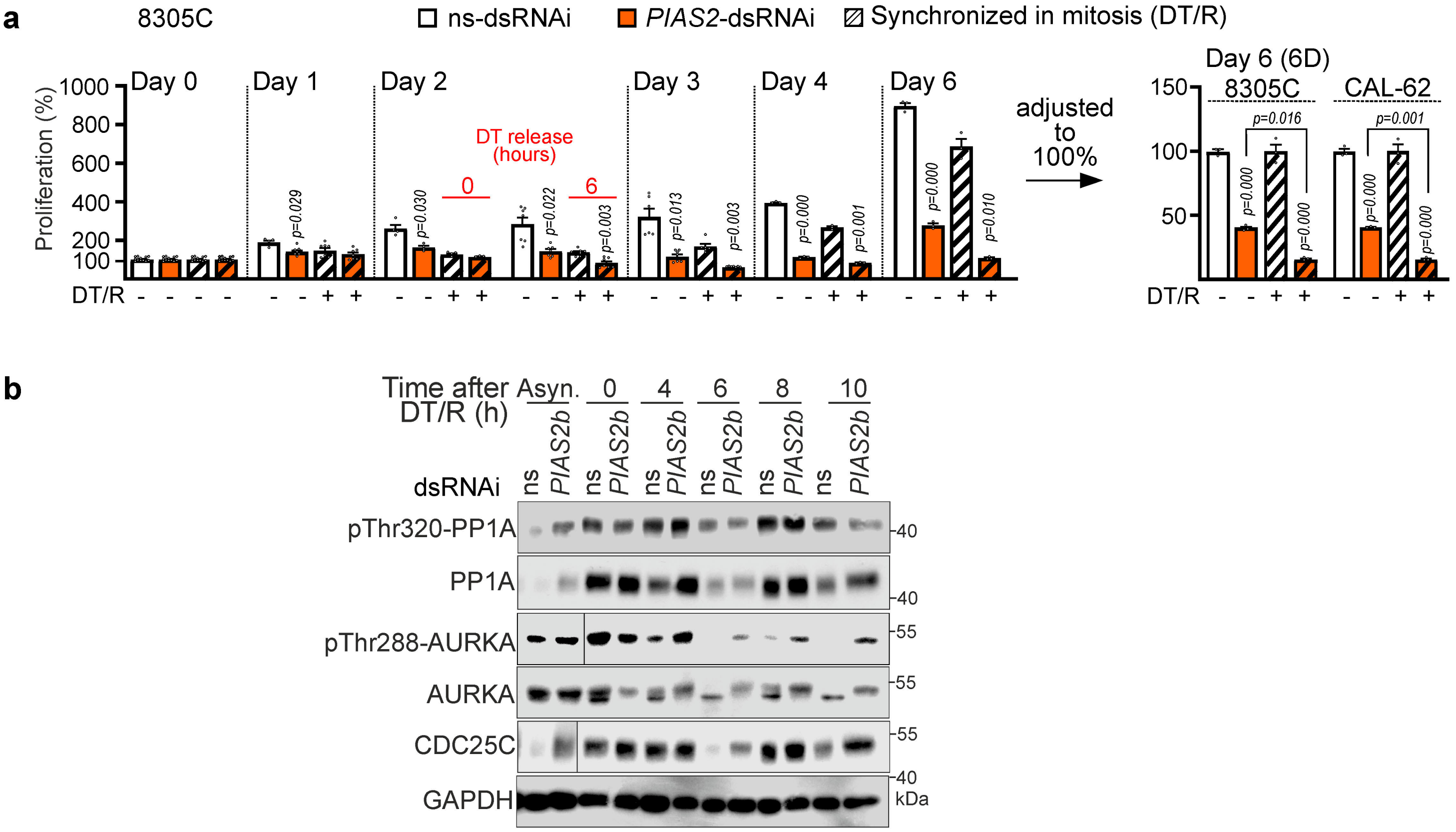

Supplementary Figure 6. PIAS2b role in mitosis is essential in ATC: mitotic protein alterations with *PIAS2b*-dsRNAi. **(a)** Cell growth assays following the designed protocol time-course (Cartoon Figure 4a), comparing asynchronous or DT/R synchronized cells. *PIAS2b*-dsRNAi has a significantly greater anti-cancer effect in mitotic synchronized than in asynchronous 8305C and CAL-62 ATC cells. **(b)** From DT/R-4h, AURKA phosphorylation, and specifically at Thr-288 AURKA phosphorylation was highly enhanced in *PIAS2b*-dsRNAi. CDC25C, the mitotic activating phosphatase, was also accumulated. No change is seen in total or Thr320 phosphorylated PP1A in *PIAS2b*-dsRNAi DT/R extracts. Although anaplastic cells present a high mitotic index naturally, pThr288-AURKA and CDC25C detection in Asynchronous cells needed longer exposure (spliced westerns).

(a) n = 7 (Days 1-3), n = 3 (Days 4-6) independent experiments with replicates, two-sided one-way ANOVA with Tukey's multiple comparison test. Bar indicate means ± SEM; exact p value is indicated in the figure. Source data are provided as a Source Data file.

Suppl Figure 7

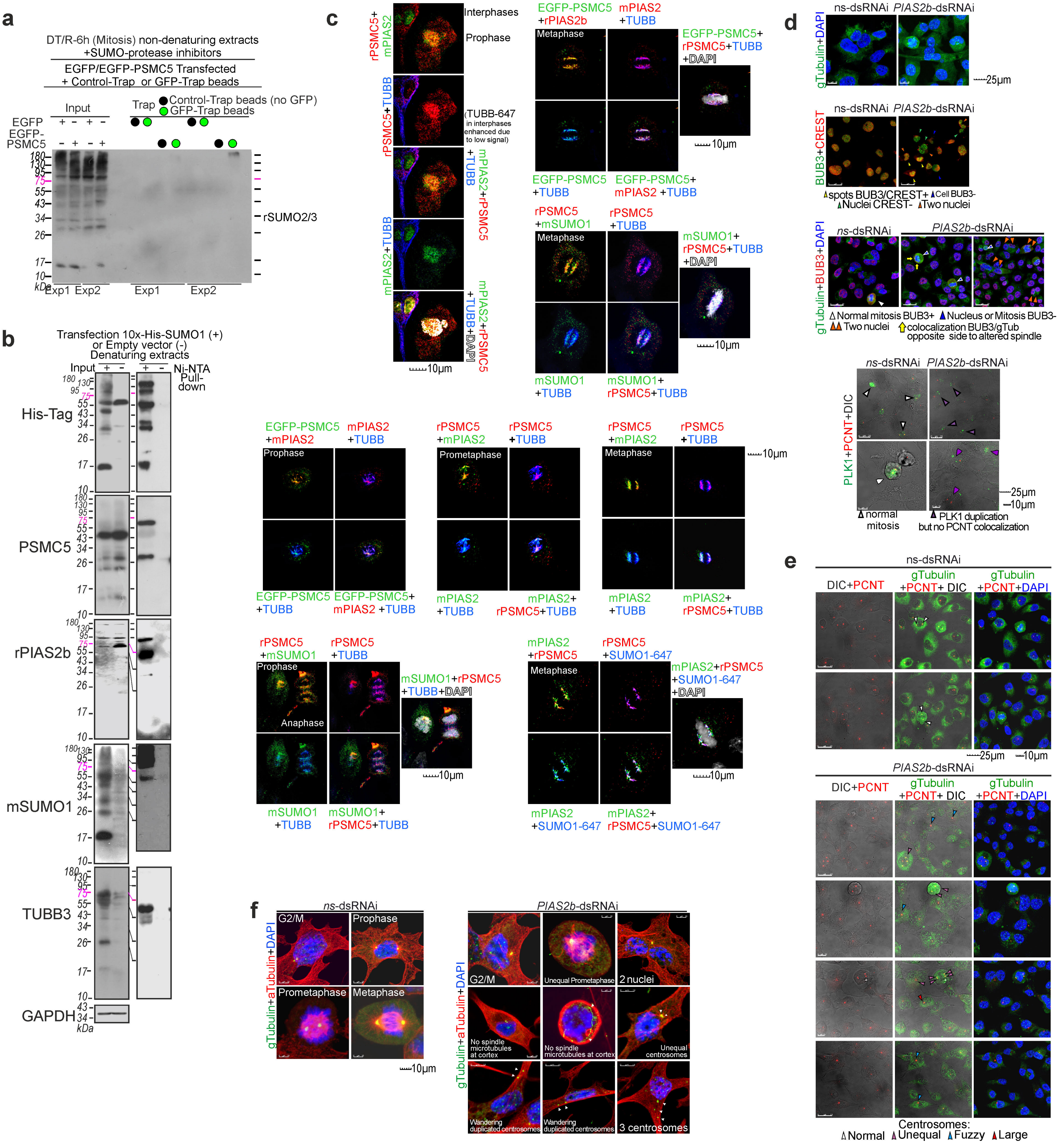

Supplementary Figure 7. PIAS2b role has an essential role in mitosis in ATC, and *PIAS2b*-dsRNAi causes mitotic perturbations at the spindle and kinetochores. (a) (Pertaining to Fig 7a, pull-down of SUMO1ylated EGFP-PSMC5 using GFP-Trap). A similar GFP-Trap with extracts obtained from cells transfected with EGFP-PSMC5 or EGFP-vector, and synchronized at mitosis (DT/R-6h), and followed by SUMO2 western blot did not give any specific band. Shown are two independent experiments. **(b)** (Pertaining to Fig 7b, standardization of Histidine-tagged SUMO1 pull downs with Nickel beads). Guanidinium-HCl denaturing extracts of cells transfected with 10x-Histidine-tagged SUMO1 or Empty vector were pull down with Nickel-beads. Western blots for indicated proteins were compared to Input extracts (no pull-down). In can be observed that in the absence of 10x-His-SUMO1, no band is shown in the Nickel beads pull-downs with any of the relevant antibodies. **(c)** (Related to main Figures 7d-7e, quantification of colocalization and 3D surface rendering study). Selected planes of confocal micropcopy cells at DT/R-6h colocalizing different markers. Combination of some of the following: EGFP-PSMC5 or rPSMC5, rPIAS2b or mPIAS2, mSUMO1 or directly labeled SUMO1-647, TUBB-647, DAPI. Mitotic phase is indicated in each group of pictures, corresponding to different channel combination. **(d-f)** Related to main Figure 7h; representative pictures of quantifications. **(d)** Mitotic perturbations at the kinetochores. From top to bottom: In *PIAS2b*-dsRNAi cells, nuclear gTubulin spots at the nucleus increase of average from 5 to 23, coincident with expected kinetochores. Nuclear gTubulin spots colocalize with BUB3 in *PIAS2b*-dsRNAi cells. Alternation of nuclei with colocalizing BUB3 plus CREST spots (yellow arrowheads, n > 3), and CREST negative nuclei (green arrowheads) in *PIAS2b*-dsRNAi treated cells. Some cells contained two nuclei (orange arrowhead), and rare BUB3 negative cells (blue arrowhead) also could be found. Duplicated PLK1 (mitosis onset) and PCNT do not colocalize in *PIAS2b*-dsRNAi treated 8305 ATC cells. **(e)** Replicated experiments showing centrosomal alterations in 8305C at mitosis (DT/R-6h) treated with *PIAS2b*-dsRNAi but not when treated with ns-dsRNAi. Colocalization of tubulin-gamma (gTubulin) and pericentrin (PCNT) reveals qualitative (unequal, fuzzy) and quantitative (< 2, > 3) centrosome alterations. **(f)** Mitotic perturbations of the spindle found at DT/R-5 h in PIAS2b-dsRNAi treated 8305 ATC cells, revealed by aTubulin and gTubulin co-staining. Source data are provided as a Source Data file.

Suppl Figure 8

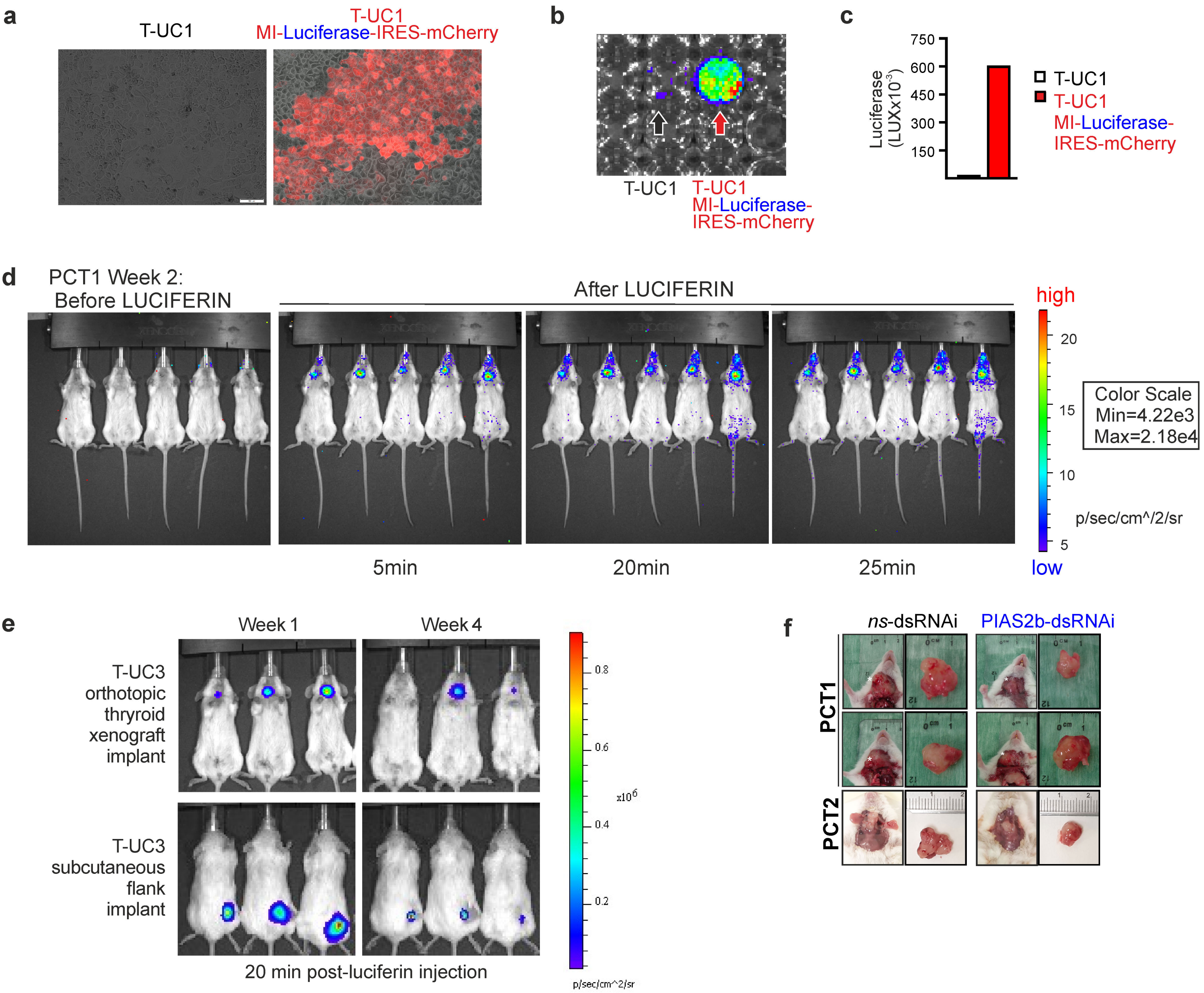

Supplementary Figure 8. Standardization of the orthotopic patient-derived xenograft (oPDX) model. Fresh tumor dissection shows macroscopic reduced volume after *PIAS2b*-dsRNAi treatment in Preclinical Trials. (a) Patient cultured cells were infected with a lentivirus expressing Luciferase-IRES-mCherry. Shown the T-UC1 culture. **(b)** Luciferase was added in one well of parental T-UC1, and in the T-UC1 infected with the lentivirus. The plate was observed at the IVIS device. **(c)** Extraction of the wells and further quantification in the luminometer. **(d)** Five mice with oPDX from T-UC1 at week 2 after injection. Signal before i.p. luciferine injection, and a time-course of detection at the IVIS. **(e)** T-UC3 did not grow in nude mice (shown are NSG) neither when implanted as orthotopic thyroid xenograft nor when implanted subcutaneously at the left flank. **(f)** Animals at endpoint of two of the Pre-Clinical (PCT) trials, showing the oPDX cancer at the neck. PIAS2b-dsRNAi tumors show visibly reduced volumes. Macroscopic dissection of the tumors show reduction, in spite of the cancers were extended and they presented appreciably difficulties for dissection.

Suppl Figure 9

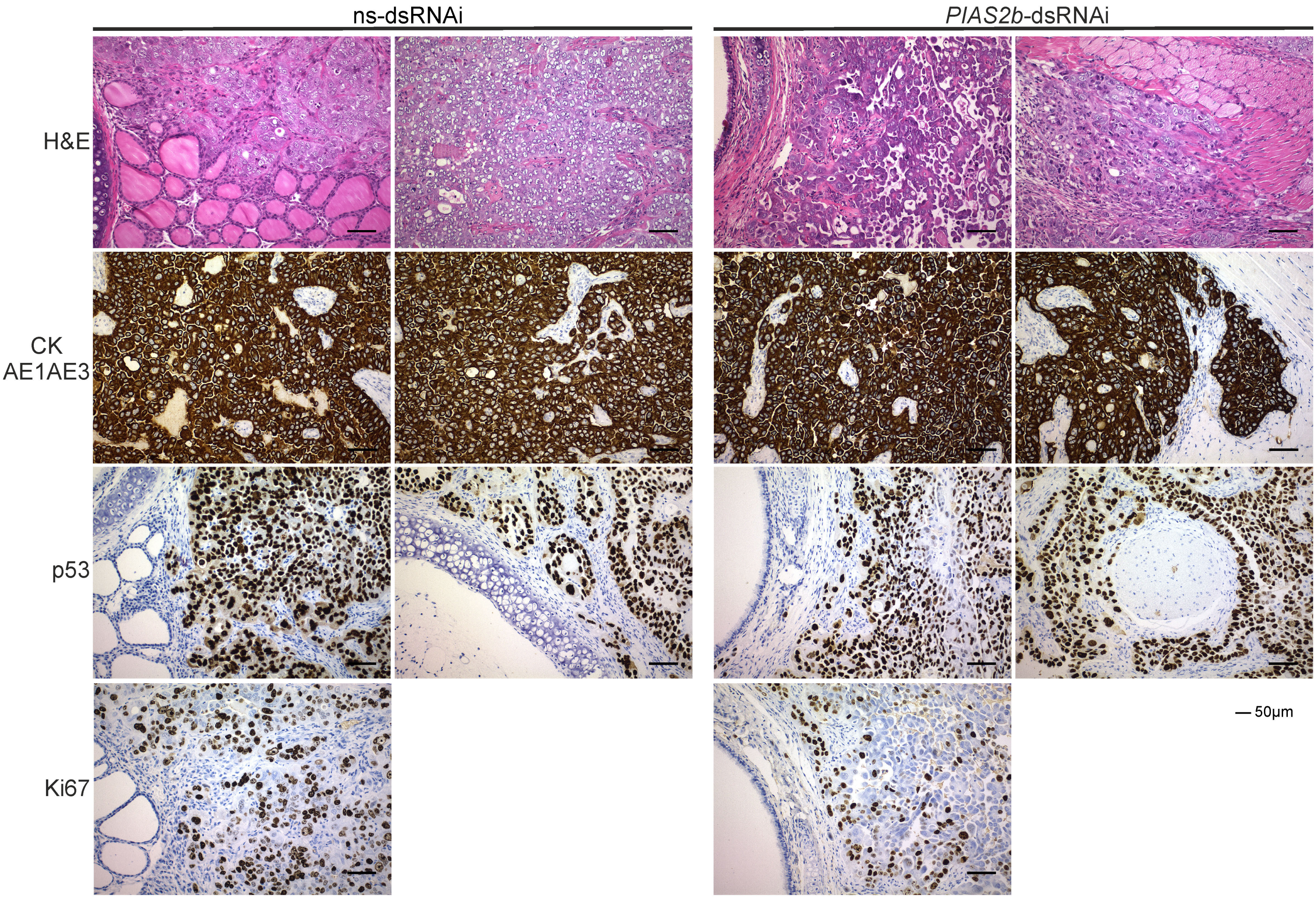

Supplementary Figure 9. Reproducible pathological analysis using clinical markers in oPDX. (Related to main Figure 9). Two other oPDX tumors from each treatment, ns-dsRNAi and *PIAS2b*-dsRNAi, stained with H&E showing the aggressive invasive behavior for thyroid and local structures. Immunohistochemistry of markers of clinical pathology classified all oPDX as ATCs, and did not reveal differences with treatment. Tumors were intensively positive for cytokeratins (CK, AE1-AE3) and p53 (specific human detection). Ki67 index was very high (as expected) in ATC. No differences were found with treatment.

Suppl Figure 10

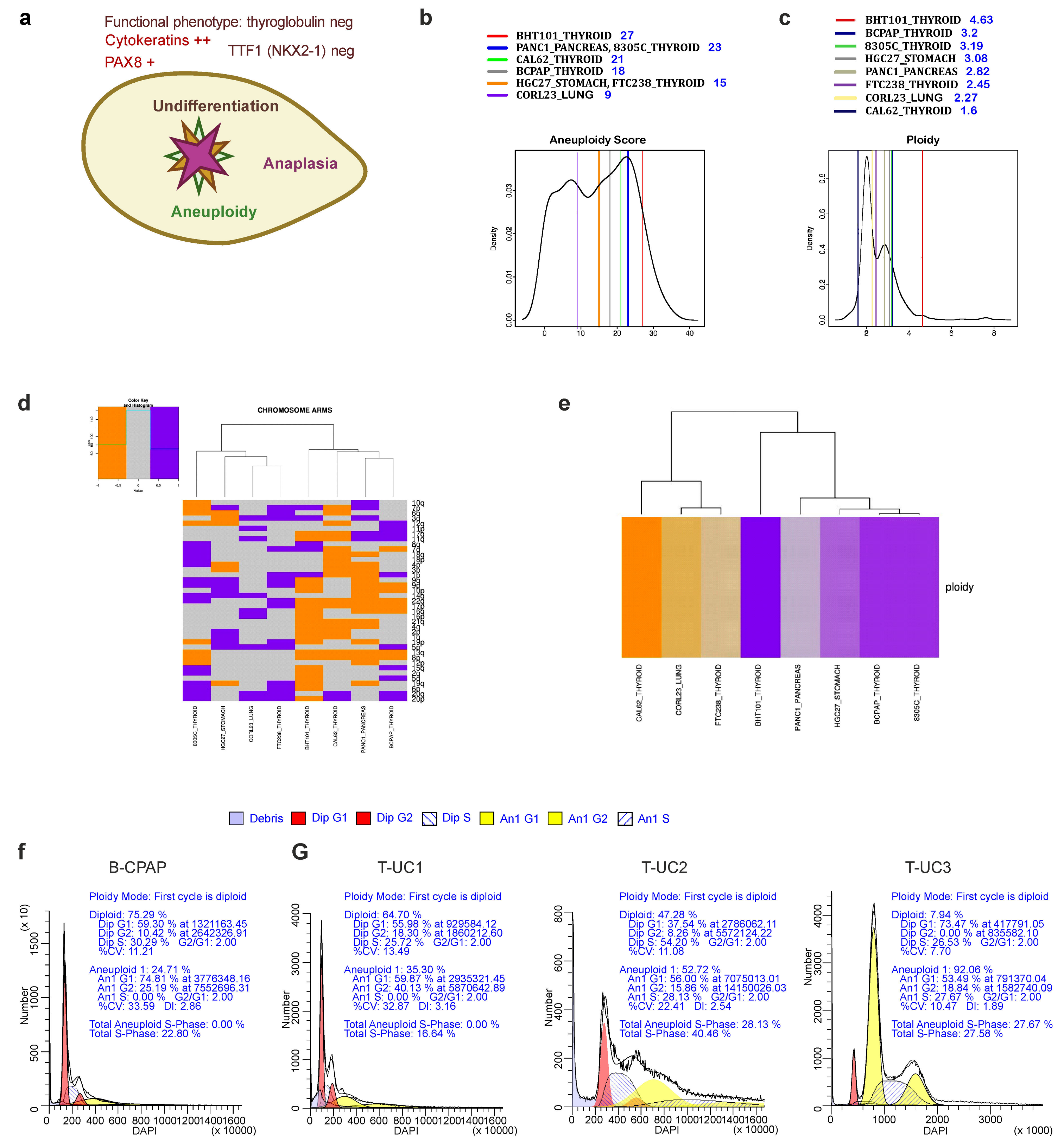

Supplementary Figure 10. *PIAS2b*-dsRNAi has anti-cancer effect in cells that combine three characteristics: Undifferentiation, Anaplasia and Aneuploidy. Aneuploidy seems to be the less important. (a) Cartoon representing the three common characteristics present in the ATC and non-thyroid anaplastic cells where *PIAS2b*-dsRNAi has anti-cancer effect: i) Undifferentiation, or absence of characteristic phenotypic markers of the original epithelium. In ATC all are Cytokeratin positive epithelial cells; but they lose TTF1 (NKX2-1) and thyroglobulin, together with total or partial loss of PAX8; ii) Anaplasia, cellular atypia with pleomorphic features; iii) Aneuploidy, higher or lower than 2n genetic content together with polyploidy in coexisting cell populations. **(b-e)** Bioinformatic data retrieved from the seminal study of Coher-Sharir Y, et al., Nature 2021 in which Aneuploidy was defined molecularly with deep sequencing in a panel of 1,000 human cancer cell lines (tabulated in Table S2). Data were available for the following cell lines used in this study: anaplastic thyroid carcinoma (BHT-101, 8305C, CAL-62); anaplastic non-thyroid carcinomas (PANC-1; COR-L23; HGC-27); poorly-differentiated thyroid carcinoma of papillary origin (B-CPAP); metastatic follicular thyroid carcinoma (FTC-238). **(b)** Quantitative aneuploidy score, and position of each line respect to the scores of the whole panel. Although three anaplastic lines are considered highly aneuploidy, the rest are considered intermediate with the smaller score belonging to an anaplastic cell line (COR-L23). **(c)** Ploidy respect to 2n (normal), and position of each line respect to the scores of the whole panel. All the cancer cell lines present altered ploidy being the highest and the lowest anaplastic cell lines. The second most altered is a non-anaplastic cell line (B-CPAP). The most similar to normality is an anaplastic line (COR-L23). **(d)** Heat-map study showing gain and loss of chromosome arms to look for shared abnormalities in the anaplastic cell lines. The non-anaplastic FTC-238 groups with anaplastic COR-L23, HGC-27 and 8305C; and the non-anaplastic B-CPAP groups with anaplastic PANC-1, CAL-62 and BHT-101. **(e)** No single shared gain/loss was found, nor was any association of gain/loss to ploidy common to all anaplastic cell lines. **(f-g)** Flow cytometry analysis of aneuploidy with ModFit, comparing the non-anaplastic cell line B-CPAP **(f)**, and three primary cultures established from ATC patients (T-UC1, T-UC2, T-UC3) **(g)**. The program considers T-UC3 highly aneuploidy (> 92%), T-UC2 in the middle (~52%), but there is not much difference between T-UC1 (~ 35%) and B-CPAP (24.7%).

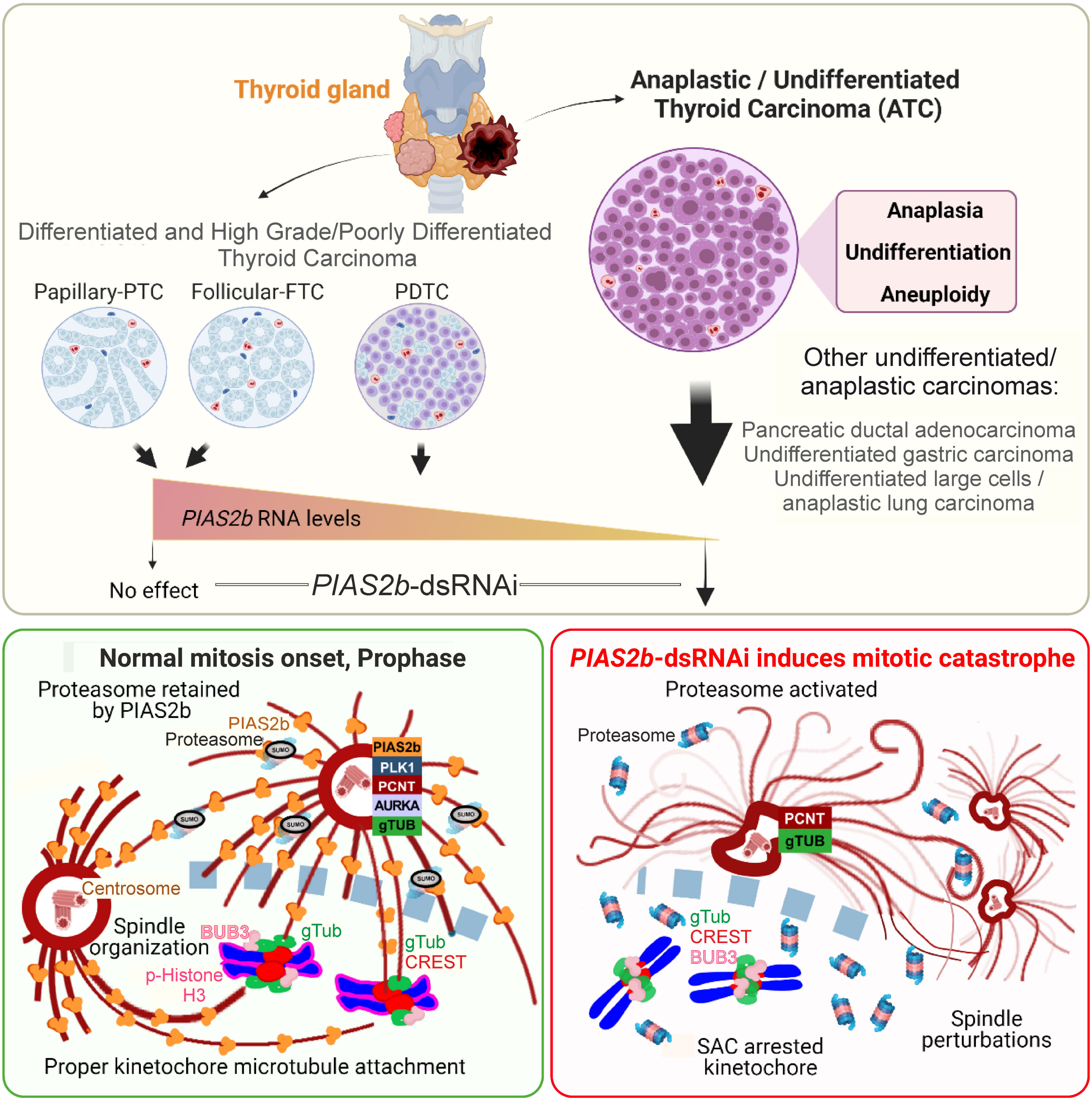

Supplementary Figure 11. Graphical Abstract. PIAS2b is a dosage-sensitive protein in Anaplastic Thyroid Carcinoma (ATC) and has an essential role at the mitotic spindle. RNA interference using in vitro transcribed, double-strand RNA against the mRNA of the PIAS2 isoform beta (*PIAS2b*-dsRNAi) kills ATC cells growing in full medium in vitro through mitotic catastrophe. Non-thyroid carcinoma cells with the same three characteristics- anaplastic, aneuploid, and undifferentiated- are also killed by *PIAS2b*-dsRNAi. *PIAS2b*-dsRNAi has no effect in Differentiated or High Grade / Poorly Differentiated Thyroid Carcinomas.
A molecular mechanism for *PIAS2b*-dsRNAi in the anaplastic carcinomas implicates untimely proteasome activation, and centrosome / spindle alterations in cell death. Thus, PIAS2b is an essential mitotic protein in anaplastic cancers that could be targeted by RNAi therapies.

NCOMMS-22-31170
dsRNAi-mediated silencing of *PIAS2beta* specifically kills anaplastic carcinomas by mitotic catastrophe

Joana S Rodrigues[1#*], Miguel Chenlo[#1], Susana B Bravo[2], Sihara Perez-Romero[1], Maria Suarez-Fariña[1], Tomas Sobrino[3], Rebeca Sanz-Pamplona[4], Roman Gonzalez-Prieto[5], Manuel Narciso Blanco Freire[6], Ruben Nogueiras[7], Miguel Lopez[8], Laura Fugazzola[9], José Manuel Cameselle-Teijeiro[10*,] Clara V Alvarez[1]

Source Data : uncropped and unprocessed scans for Supplementary figures

Suppl Fig 1e

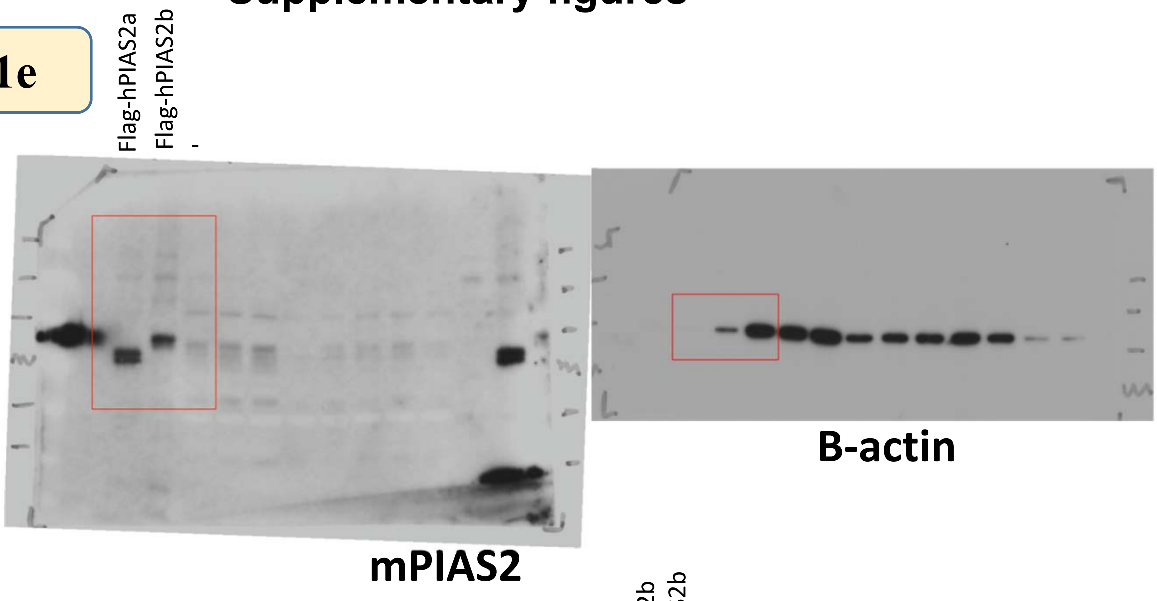

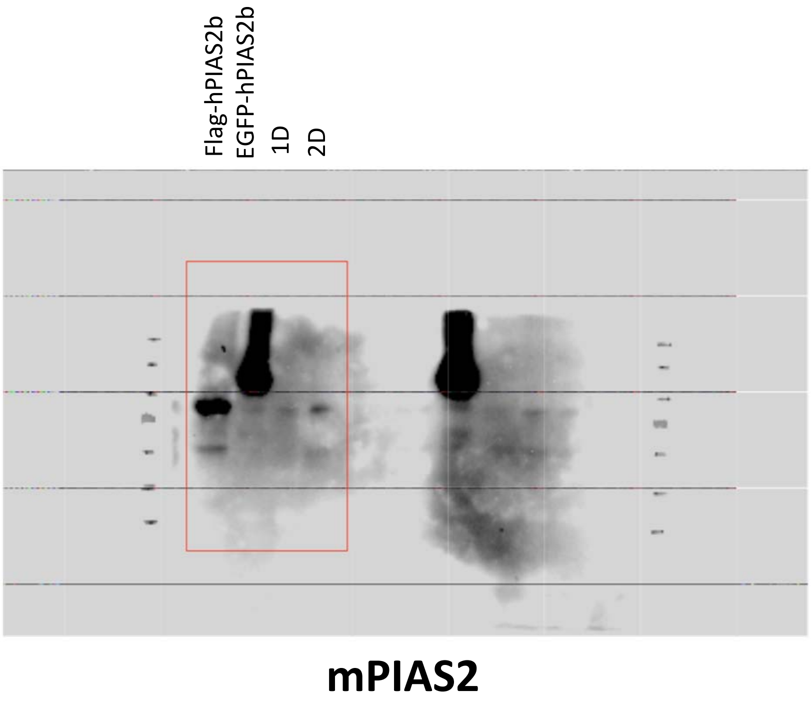

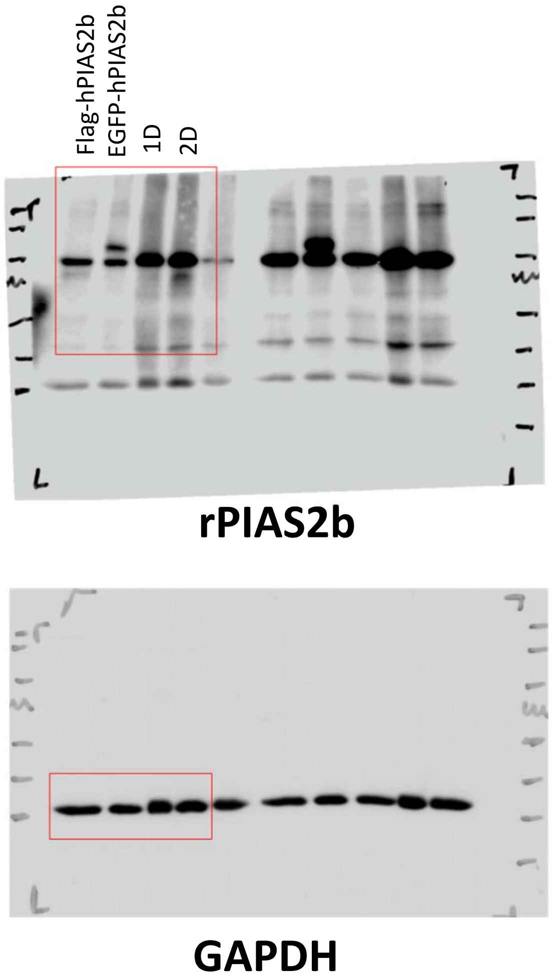

Suppl Fig 1h

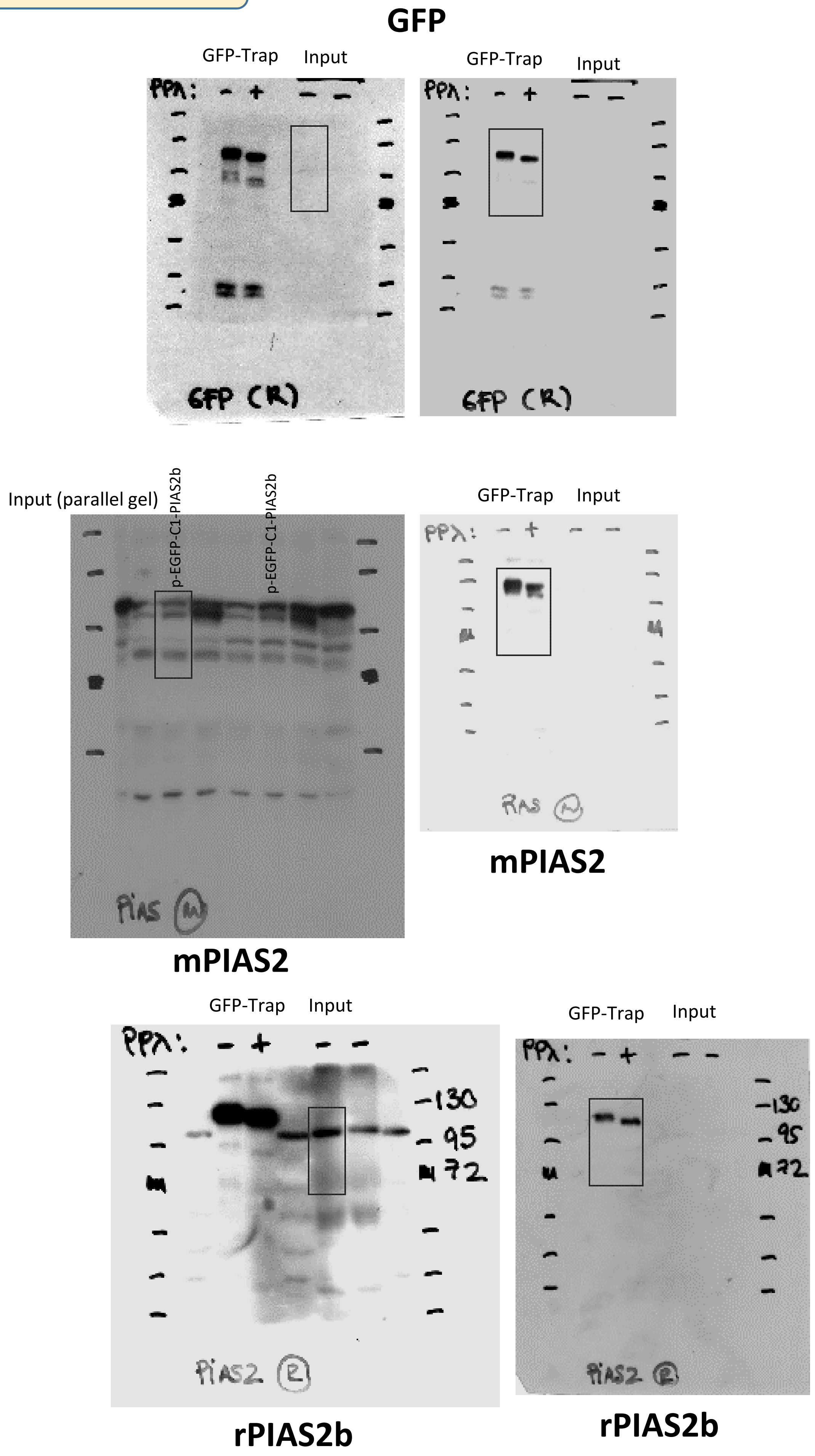
GFP
GFP-Trap
Input
GFP-Trap
Input
Input (parallel gel)
p-EGFP-C1-PIAS2b
p-EGFP-C1-PIAS2b
GFP-Trap
Input
mPIAS2
mPIAS2
GFP-Trap
Input
GFP-Trap
Input
-130
- 95
72
rPIAS2b
rPIAS2b

Suppl Fig 2d

mPIAS2

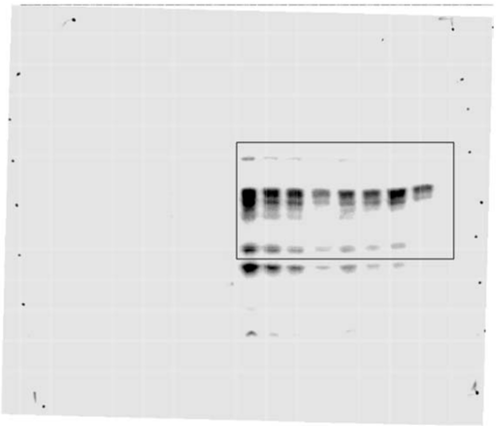

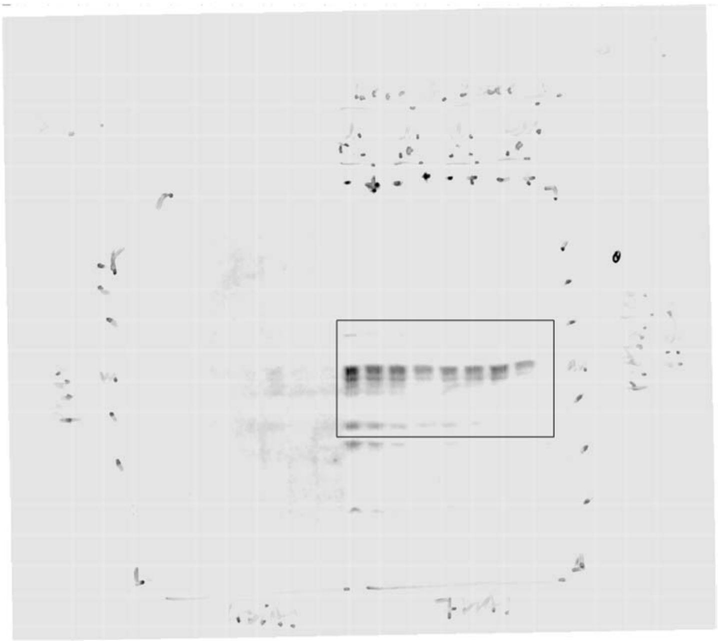

mPIAS2

Beta-Actin

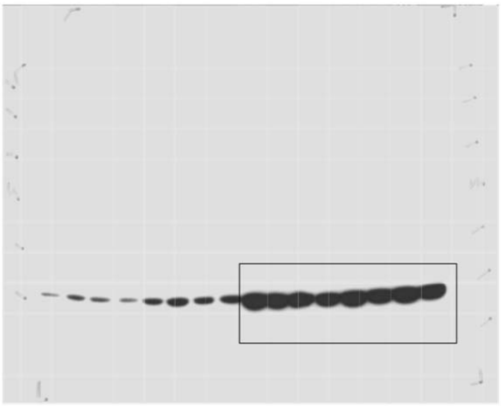

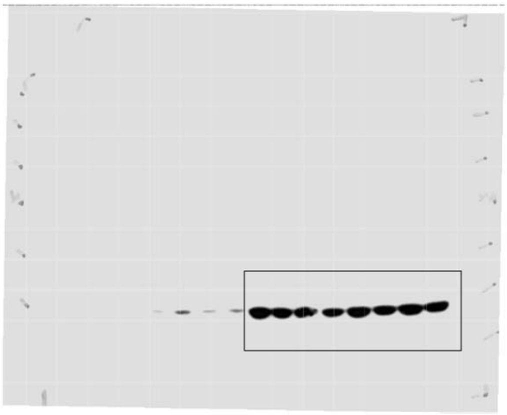

GAPDH

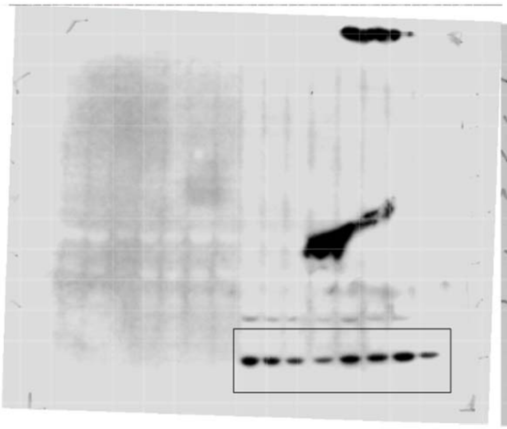

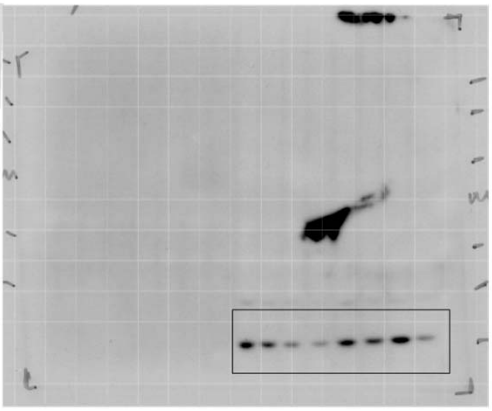

Suppl Fig 5a

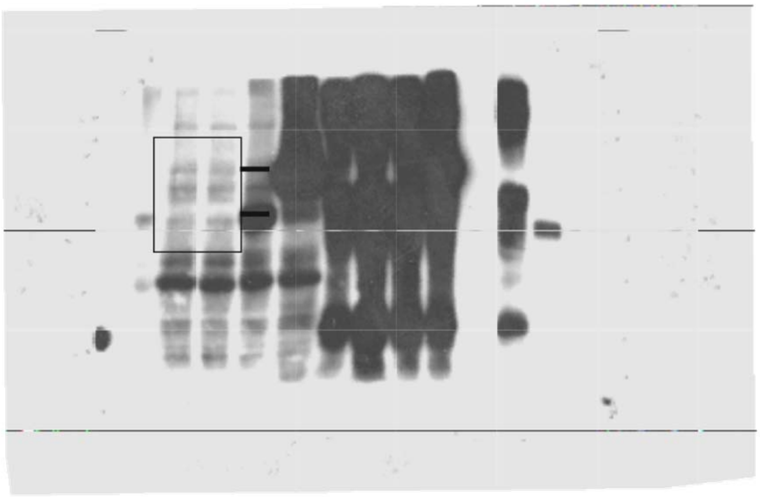

mPIAS2

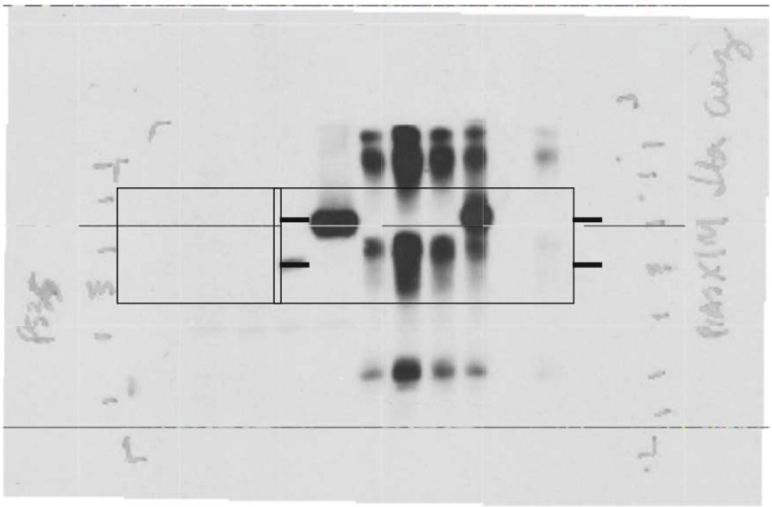

mPIAS2

aTUBULIN

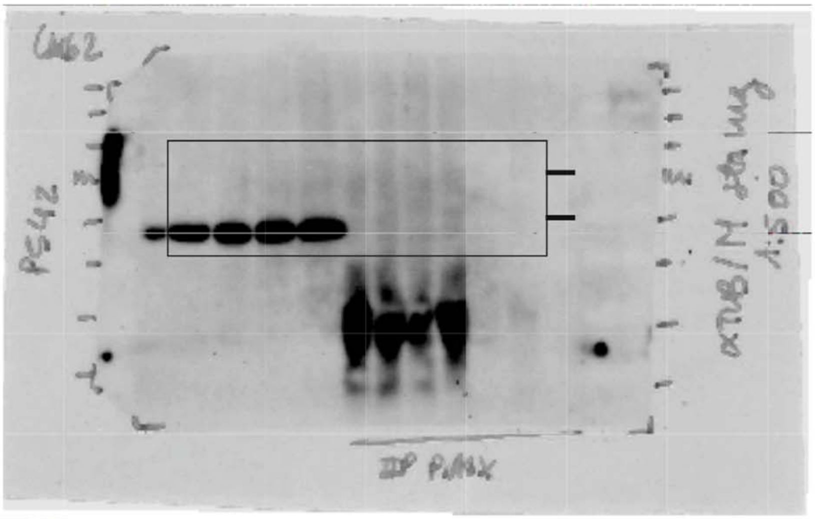

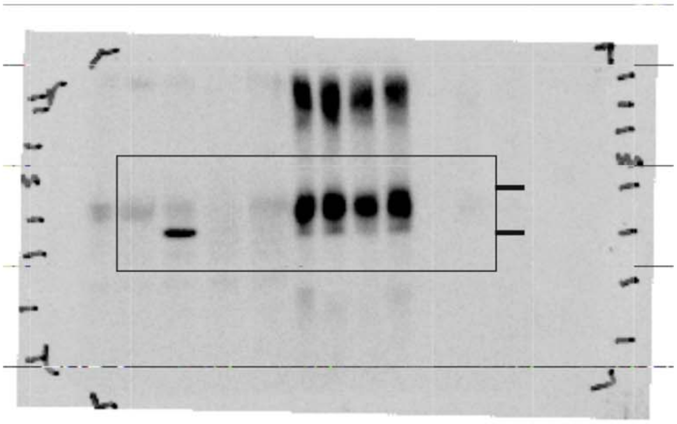

AURKA

Suppl Fig 6b

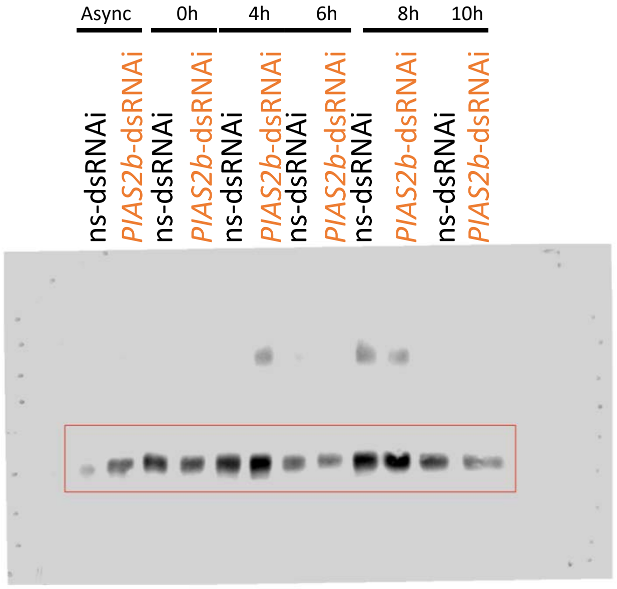

pThr320-PP1A

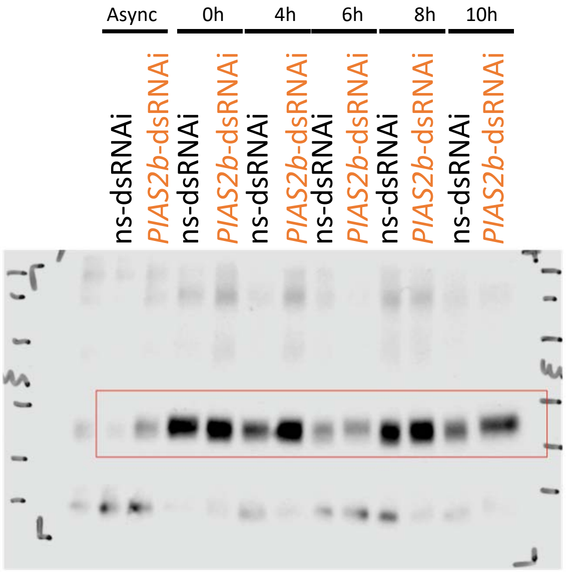

PP1A

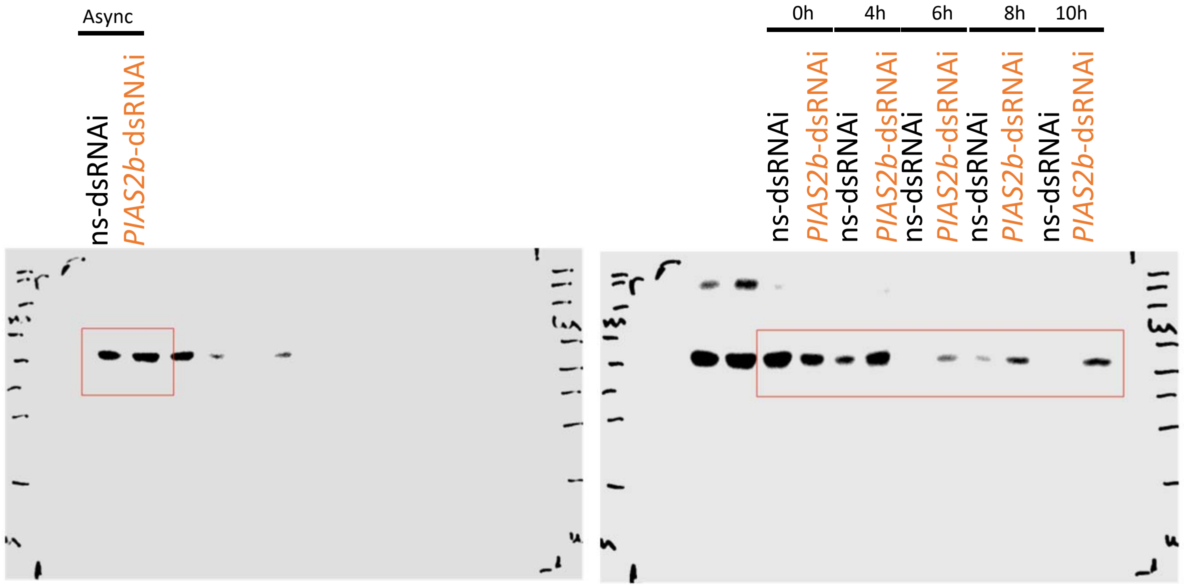

pThr288-AURKA

Suppl Fig 6b

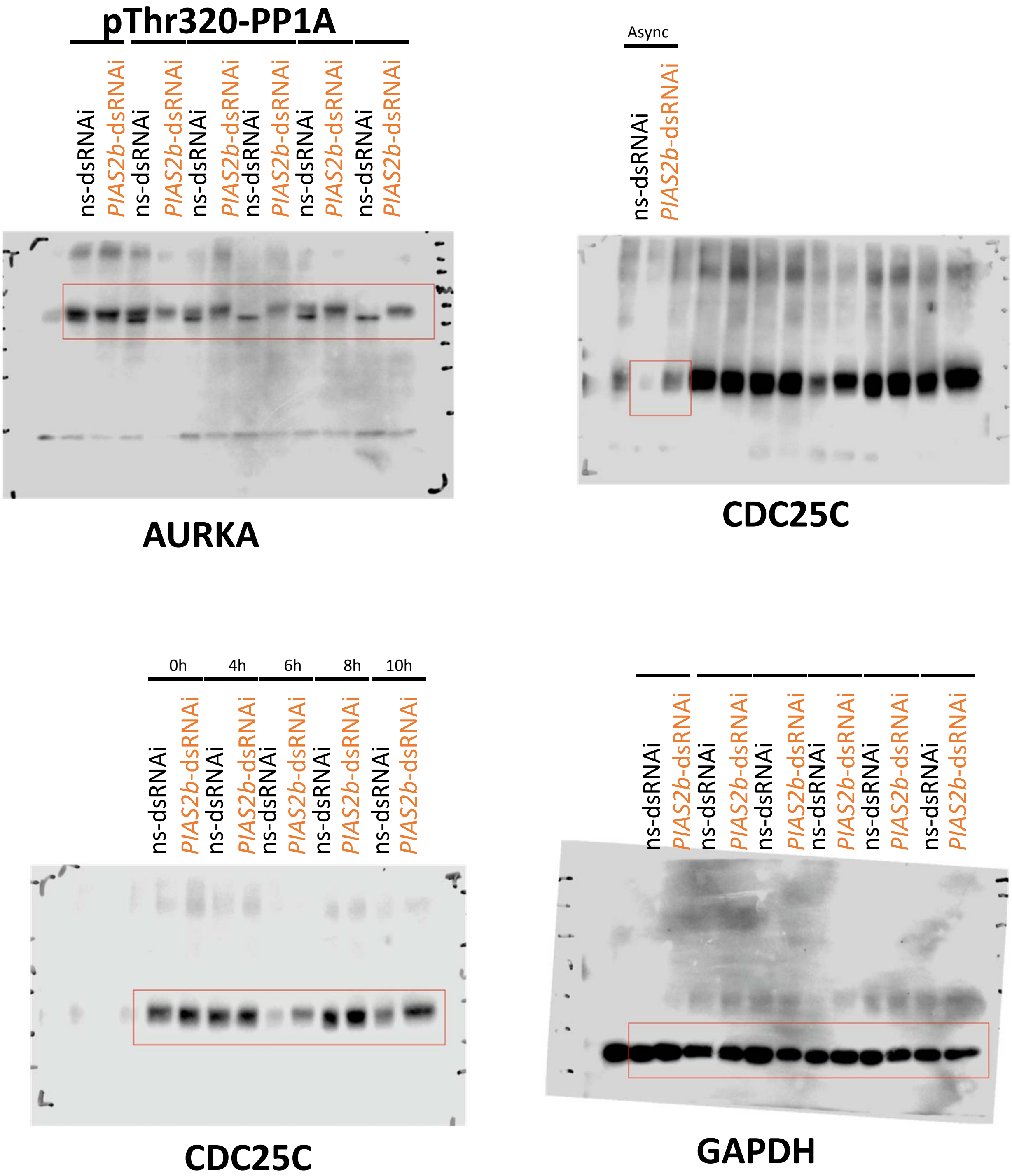
pThr320-PP1A
ns-dsRNAi
PIAS2b-dsRNAi
AURKA
Async
CDC25C
0h
4h
6h
8h
10h
CDC25C
GAPDH

Suppl Fig 7a

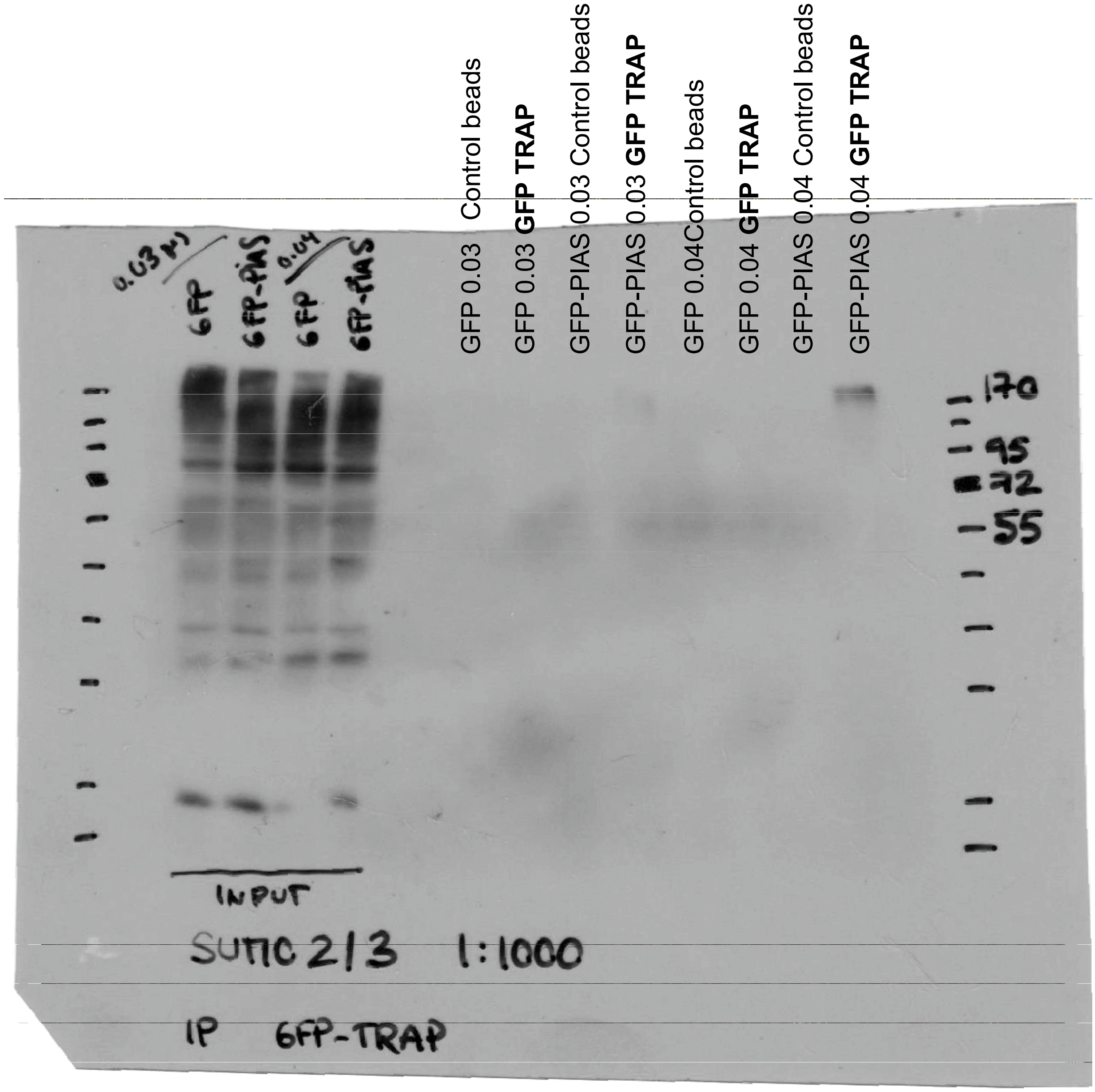
GFP 0.03 Control beads
GFP 0.03 GFP TRAP
GFP-PIAS 0.03 Control beads
GFP-PIAS 0.03 GFP TRAP
GFP 0.04Control beads
GFP 0.04 GFP TRAP
GFP-PIAS 0.04 Control beads
GFP-PIAS 0.04 GFP TRAP
0.03 µl
GFP
GFP-PIAS
0.04
GFP
GFP-PIAS
170
95
72
55
INPUT
SUMO 2/3 1:1000
IP GFP-TRAP

Suppl Fig 7b

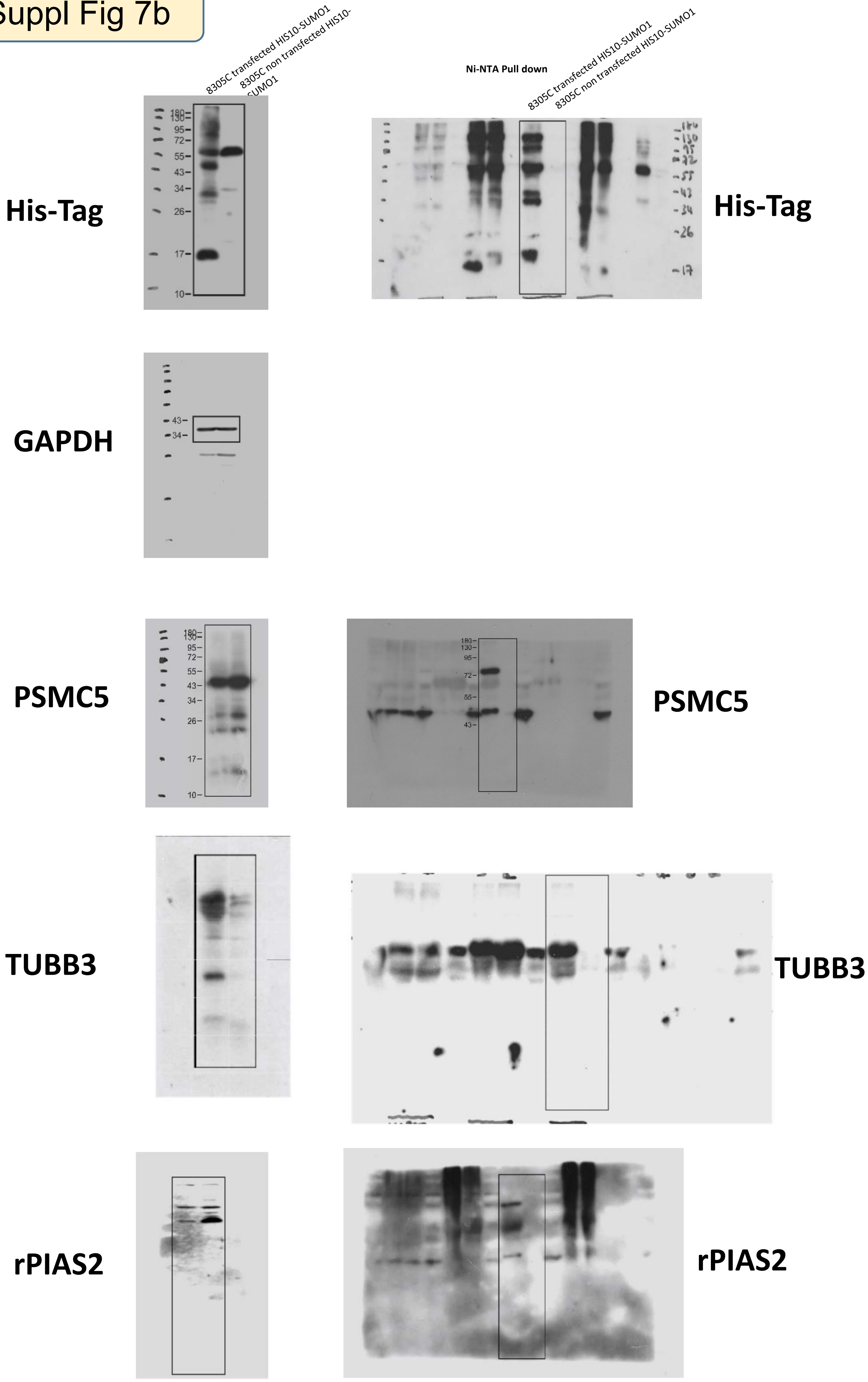

8305C transfected HIS10-SUMO1
8305C non transfected HIS10-SUMO1
Ni-NTA Pull down
8305C transfected HIS10-SUMO1
8305C non transfected HIS10-SUMO1
His-Tag
His-Tag
GAPDH
PSMC5
PSMC5
TUBB3
TUBB3
rPIAS2
rPIAS2
