# Supplementary material for: dsRNAi-mediated silencing of PIAS2beta specifically kills anaplastic carcinomas by mitotic catastrophe
Source: Nat Commun. 2024 May 14;15:3736. doi: 10.1038/s41467-024-47751-1 (PMC11094195; doi:10.1038/s41467-024-47751-1)
Supplement: Supplementary file 2 — Reporting Summary [file 41467_2024_47751_MOESM2_ESM.pdf]

nature portfolio

Corresponding author(s): Jose Manuel Cameselle-Teijeiro
Clara V Alvarez

Last updated by author(s): Apr 9, 2024

Reporting Summary

Nature Portfolio wishes to improve the reproducibility of the work that we publish. This form provides structure for consistency and transparency in reporting. For further information on Nature Portfolio policies, see our Editorial Policies and the Editorial Policy Checklist.

Statistics

For all statistical analyses, confirm that the following items are present in the figure legend, table legend, main text, or Methods section.

n/a	Confirmed	
☐	☒	The exact sample size (*n*) for each experimental group/condition, given as a discrete number and unit of measurement
☐	☒	A statement on whether measurements were taken from distinct samples or whether the same sample was measured repeatedly
☐	☒	The statistical test(s) used AND whether they are one- or two-sided<br>*Only common tests should be described solely by name; describe more complex techniques in the Methods section.*
☒	☐	A description of all covariates tested
☐	☒	A description of any assumptions or corrections, such as tests of normality and adjustment for multiple comparisons
☐	☒	A full description of the statistical parameters including central tendency (e.g. means) or other basic estimates (e.g. regression coefficient) AND variation (e.g. standard deviation) or associated estimates of uncertainty (e.g. confidence intervals)
☐	☒	For null hypothesis testing, the test statistic (e.g. *F*, *t*, *r*) with confidence intervals, effect sizes, degrees of freedom and *P* value noted<br>*Give P values as exact values whenever suitable.*
☒	☐	For Bayesian analysis, information on the choice of priors and Markov chain Monte Carlo settings
☒	☐	For hierarchical and complex designs, identification of the appropriate level for tests and full reporting of outcomes
☒	☐	Estimates of effect sizes (e.g. Cohen's *d*, Pearson's *r*), indicating how they were calculated

Our web collection on statistics for biologists contains articles on many of the points above.

Software and code

Policy information about availability of computer code

Data collection	Flow citometry: BD Accuri™ C6 cytometer (BD Biosciences) using the violet laser (405 nm).<br>Time-lapse imaging: Leica microscope (CTR7000 HS, Leica);<br>LC-MS/MS proteomic assays: Triple-TOF 6600 (SCIEX) system using a data-dependent workflow (DDA) ;<br>Immunofluorescence microscopy: confocal microscope (Leica TC SP5-AOBS) with white laser (470-670nm) and ultraviolet laser, through the 63x oil objective with numerical aperture (NA) 1.4 and LAS AF software (Leica Application Suite Advanced Fluorescence;<br>Proteasome activity assay: luminometer (Multimode Mithras LB 940, Berthold Technologies);<br>SkanIt™ Software for Microplate Readers (Thermo Scientific)<br>in vivo imaging system: IVIS Spectrum imaging system, Perkin Elmer.
Data analysis	The list of Software used in this study are as follows:<br>Living Image v3.2;<br>FlowJo software v10;<br>ModFit LT v5;<br>Leica LASX program v3.3;<br>Block-iT siRNA Designer;<br>Regulatory SNP (rSNPs) MAPPER;<br>PDQuest program v8.0.1;<br>Progenesis SameSpots v4.5;<br>Mascot software v2.1;

Analyst TF V1.7.1;
ProteinPilot v5.0.2 ;
Cytoscape v3.6.1;
STRING v11;
FunRich v 3.1.3;
Automated Cellular Imaging System (ACIS III);
FiJi program (ImageJ 1.52);
R Project for Statistical Computing;
GraphPad Prism v8.0.1;
SPSS Statistics v20;
Corel Draw Graphic Suite 2017;
BioRender
SkanIt
cellSens standard (v4.1)
Scaffold (v5.2)

For manuscripts utilizing custom algorithms or software that are central to the research but not yet described in published literature, software must be made available to editors and reviewers. We strongly encourage code deposition in a community repository (e.g. GitHub). See the Nature Portfolio guidelines for submitting code & software for further information.

Data

Policy information about availability of data

All manuscripts must include a data availability statement. This statement should provide the following information, where applicable:

- Accession codes, unique identifiers, or web links for publicly available datasets
- A description of any restrictions on data availability
- For clinical datasets or third party data, please ensure that the statement adheres to our policy

Source Data provided for each section is indicated in the Figure legends.

Publicly available datasets:
Cellosaurus v31; https://www.cellosaurus.org/index.html
Misidentified Cell Lines v9; https://www.atcc.org/en/services/cell-authentication?gad_source=1&gclid=EAIaIQobChMI--r6hPLhhAMVB5mDBx2lLQGWEAAYASAAEgKi1vD_BwE
NCBI BLAST; https://blast.ncbi.nlm.nih.gov/Blast.cgi
NCBI database;https://www.ncbi.nlm.nih.gov/
Ensembl;https://www.ensembl.org/
Uniprot database;https://www.uniprot.org/
GPS-SUMO 2.0; https://sumo.biocuckoo.cn/
TCGA datasets Broad Institute portal http://gdac.broadinstitute.org, obtained through the HUMAN PROTEIN ATLAS consortium https://www.proteinatlas.org/ENSG00000078043-PIAS2/pathology
Aneuploidy scores and Ploidy scores obtained from Source Data of Cohen-Sharir Y, et al, & Ben-David U. Aneuploidy renders cancer cells vulnerable to mitotic checkpoint inhibition. Nature. 2021 Feb;590(7846):486-491. doi: 10.1038/s41586-020-03114-6. Epub 2021 Jan 27. PMID: 33505028; PMCID: PMC8262644.
The mass spectrometry proteomics raw data have been deposited to the ProteomeXchange Consortium via the PRIDE 107 partner repository with the dataset identifier PXD044110. https://www.ebi.ac.uk/pride/

Research involving human participants, their data, or biological material

Policy information about studies with human participants or human data. See also policy information about sex, gender (identity/presentation), and sexual orientation and race, ethnicity and racism.

Reporting on sex and gender

TIROCHUS collection is active from 2012, and recruits consecutively all patients with thyroid surgery for any benign or tumor disease. Fresh tissue together with age and sex, and diagnosed clinical pathology after surgery are collected consecutively along the year. Some patients generate 2-4 samples from indepedent lesions after the thyroidectomy. Al the moment TIROCHUS has more than 1,300 samples, 600 independent patients, >70% are women, reflectin the epidemiology of the thyroid tumor pathology.

Reporting on race, ethnicity, or other socially relevant groupings

Please specify the socially constructed or socially relevant categorization variable(s) used in your manuscript and explain why they were used. Please note that such variables should not be used as proxies for other socially constructed/relevant variables (for example, race or ethnicity should not be used as a proxy for socioeconomic status).
Provide clear definitions of the relevant terms used, how they were provided (by the participants/respondents, the researchers, or third parties), and the method(s) used to classify people into the different categories (e.g. self-report, census or administrative data, social media data, etc.)
Please provide details about how you controlled for confounding variables in your analyses.

Population characteristics

TIROCHUS is a registered collection at the ISCIII national bank with the number nº c.0003960 (to JMC-T). The patients' samples are collected consecutively without exception as blind and coded. Sex and age are recorded with each sample, and two weeks later, the clinical pathology diagnosis is adjoined. Our collection is >75% women as corresponding to the described epidemiology of thyroid tumor pathology.
Some years ago we obtained standardized conditions for growing in vitro thyroid cells from normal, benign proliferative disease and cancers in similar growing conditions (Bravo 2013 PMID: 23539720; Bravo 2010 PMID: 20122891). First, we

	established a protocol of donation of the tissue surplus from thyroid surgeries: TIROCHUS. The protocol establishes that for each patient's fragment, a culture will be established but also RNA/DNA from a fragment of the initial fresh tissue will be extracted. This will serve to genetically identify each culture with the original tissue, but also to have RNA/DNA from large successive series of cancer/benign cases. Secondly, we obtained the h7H defined medium where all components, associated in 7 groups, -osmoles, hormones, energetic nutrients, iodine, antioxidants, oligoelements, and ethanol-, were carefully adjusted to concentrations found in normal human serum. In the group of hormones, those having a demonstrated action in thyroid cells were added. The cultures are 95% epithelial (Citokeratin+) and maintain the phenotype demonstrated by staining for expression of thyroid-specific transcription factors (NKX2.1 or TTF1, PAX8) and proteins (Tg, TPO) but also by measuring Tg and thyroid hormone secretion into the culture medium. We have obtained relevant results with h7H explaining pathophysiology of thyroid cancer, correlating in vitro functional data to in vivo tissue data (Garcia-Rendueles 2017. PMID: 27452523).
Recruitment	We are a multidisciplinary team. Informed consent and protocols, approved by the ethical committee (see below section), are presented to the patient by the surgeon, with unbiased explanation about TIROCHUS goals, and destiny of surplus surgical samples. >95% patients sign generously their consect.
Ethics oversight	The study has the approval of the regional committee on ethics in clinical research (Comité Ético de Investigación Clínica de Galicia, CEIC, Servizo Galego de Saúde, SERGAS), and is registered with the number 2016/239 and 2019/533 (to CVA). TIROCHUS is a registered collection at the ISCIII national bank with the number nº c.0003960 (to JMC-T). Informed consent was obtained from each patient with a specific signed document updated to the current legislation. The sample series from Italy were approved by the Ethical Committee of the IRCCS Istituto Auxologico Italiano (#2018_09_25_04 to LF).

Note that full information on the approval of the study protocol must also be provided in the manuscript.

Field-specific reporting

Please select the one below that is the best fit for your research. If you are not sure, read the appropriate sections before making your selection.

☒ Life sciences ☐ Behavioural & social sciences ☐ Ecological, evolutionary & environmental sciences

For a reference copy of the document with all sections, see nature.com/documents/nr-reporting-summary-flat.pdf

Life sciences study design

All studies must disclose on these points even when the disclosure is negative.

Sample size	Sample size calculation was obtained the calculator of Prof. Weyne W LaMorte (Boston University, https://sphweb.bumc.bu.edu/otlt/MPH-Modules/Excel/Excel6.html) using previous known data.<br>For in vitro experiments Sample Size was calculated as follows: The Standard Deviation (SD) of proliferation assays in cell lines and primary cultures was well established in our laboratory, and less than 10% with n=8 technical replicates (see References); with this, expecting a difference in growth >35%, with a power of 90%, and a two-sided alpha level of 0.05, the sample size (minimum of independent experiments) was 3.<br>References: Garcia-Rendueles AR, et al, & Alvarez CV. Rewiring of the apoptotic TGF-β-SMAD/NFκB pathway through an oncogenic function of p27 in human papillary thyroid cancer. Oncogene. 2017 Feb 2;36(5):652-666. doi: 10.1038/onc.2016.233. Epub 2016 Jul 25. PMID: 27452523) and (Bravo SB, Garcia-Rendueles ME, Garcia-Rendueles AR, et al, & Alvarez CV. Humanized medium (h7H) allows long-term primary follicular thyroid cultures from human normal thyroid, benign neoplasm, and cancer. J Clin Endocrinol Metab. 2013 Jun;98(6):2431-41. doi: 10.1210/jc.2012-3812. Epub 2013 Mar 28. PMID: 23539720.<br><br>For Primary Cultures, the n=3 independent experiment was performed with only 3 technical replicate in each, due to the lower number of cells available.<br><br>For animal experiments, a Standard Deviation (SD) of the Luciferase signal at the IVIS was considered to be higher, at 25%. The difference in growth appreciated as different was considered > 35%. Thus sample size was calculated for a power of 80% and alpha of two-sided 0.05, and it was n=5 animals per group in each Pre-Clinical Trial (PCT).
Data exclusions	The samples were included or excluded from the analysis by prior identification of the outliers using the GraphPad Prism 8.0.1 software (San Diego, USA)<br>In animals no data were excluded from the analysis.
Replication	In vitro experiments with at least three and a maximum of eight technical replicates in each independent experiment; as stated above, the minimal number of independent experiments for statistics was n=3. No experiment was excluded, and only outlier data mathematically identified by GraphPad were excluded.
Randomization	For In vitro experiments, the distribution of the different sample groups is carried out by seeding the cells in columns inside the multiwells, while the different treatments are added vertically; or vice-versa.<br>For the microscopy quantification , at least 3 fields were randomly photographed from each of the wells that form the groups of samples.<br>Mice were randomized to treatment groups.
Blinding	Blinding was not possible for in vitro experiments but the whole manuscript was performed as contrary to the results presented, as follows. The initial data, based on 2D proteomics and in TCGA ATLAS on Differentiated Papillary Thyroid Carcinomas (PTC), suggested that the important effect would be in PTC. We were working and collecting data in all types of primary cultures of thyroid tumours and cell lines. It was not until much later, that we were able to understand that the effect of PIAS2b downregulation was in Anaplastic Thyroid Cells.

Reporting for specific materials, systems and methods

We require information from authors about some types of materials, experimental systems and methods used in many studies. Here, indicate whether each material, system or method listed is relevant to your study. If you are not sure if a list item applies to your research, read the appropriate section before selecting a response.

Materials & experimental systems

n/a	Involved in the study
☐	☒ Antibodies
☐	☒ Eukaryotic cell lines
☒	☐ Palaeontology and archaeology
☐	☒ Animals and other organisms
☐	☒ Clinical data
☒	☐ Dual use research of concern
☒	☐ Plants

Methods

n/a	Involved in the study
☒	☐ ChIP-seq
☐	☒ Flow cytometry
☒	☐ MRI-based neuroimaging

Antibodies

Antibodies used

PIASx (D-12) antibody (Called mPIAS2 within this work) WB: 1:500, I-Block™; ON 4ºC+1h RT; IF:1:25 (or if blocking with IgG2a for 1 hour, 1:50) IN 4ºC; IHC: 1:500 (PT link) Santa Cruz Biotechnology Cat# sc-166494; RRID: AB_2164215

PIAS2b polyclonal antibody (Called rPIAS2b within this work) WB: 1:1000, I-Block™; ON 4ºC+1h RT; IF:1:500; IHC: 1:2000 (PT link) Atlas Antibodies Fully Validated Cat# HPA068792; RRID: AB_2686037

Aurora A antibody WB: 1:4000, I-Block™; ON 4ºC+1h RT; IF:1:250 Abcam Cat# ab1287; RRID: AB_299491

Phospho-Aurora A (Thr288) (C39D8) Rabbit mAb antibody WB: 1:500, I-Block™; ON 4ºC+1h RT; IF:1:250 Cell Signaling Technology Cat# 3079; RRID: AB_2061481

Anti-alpha Tubulin antibody - Microtubule Marker IF:1:500 Abcam Cat# ab18251; RRID: AB_2210057

Monoclonal alpha-Tubulin antibody produced in mouse WB: 1:5000, I-Block™; ON 4ºC; IF:1:50 Sigma-Aldrich Cat# T5168; RRID: AB_477579

Mouse beta-Actin Monoclonal Antibody, Unconjugated, Clone AC-74 WB: 1:5000, I-Block™; ON 4ºC; IF:1:500 Sigma-Aldrich Cat# A5316; RRID: AB_476743

Beta3 Tubulin (TU-20) antibody (Called TUBB3 within this work) WB: 1:1000; IP:0.1µg Santa Cruz Biotechnology Cat# sc-51670; RRID: AB_630408

Gamma Tubulin antibody WB: 1:1000; IF:1:1000 Abcam Cat# ab11317; RRID: AB_297921

Gamma Tubulin (D-10) antibody WB: 1:1000, I-Block™; ON 4ºC+1h RT; IF:1:250 Santa Cruz Biotechnology Cat# sc-17788; RRID: AB_628418

Plk1 (F-8) antibody WB: 1:1000 Santa Cruz Biotechnology Cat# sc-17783; RRID: AB_628157

Pericentrin antibody IF:1:1000 Abcam Cat# ab4448; RRID: AB_304461

Recombinant Anti-Bub3 antibody [EPR5319(2)] IF:1:250 Abcam Cat# ab133699 RRID: AB_2789269

Anti-Centromere Antibody IF: Ready to use Antibodies Incorporated Cat# 15-234-0001; RRID: AB_2687472

Phospho-Histone H3 (Ser10) Antibody WB: 1:1000, BSA+0.1% Tween20; ON 4ºC+1h RT; IF:1:100, IHC:1:1000 Cell Signaling Technology Cat# 9701; RRID: AB_331535

Anti-Histone H3 Antibody, Unconjugated WB: 1:1000, I-Block™; ON 4ºC+1h RT Cell Signaling Technology Cat# 9715; RRID: AB_331563

PP2A-Calpha/beta (0.T.118) antibody WB: 1:1000, BSA+0.1% Tween20; ON 4ºC+1h RT Santa Cruz Biotechnology Cat# sc-56950; RRID: AB_1128767

p-PP2A-Calpha/beta (F-8) antibody WB: 1:500, BSA+0.1% Tween20; ON 4ºC+1h RT Santa Cruz Biotechnology Cat# sc-271903; RRID: AB_10611810

PP1 (E-9) antibody WB: 1:1000, I-Block™; ON 4ºC+1h RT Santa Cruz Biotechnology Cat# sc-7482; RRID: AB_628177

Phospho-PP1alpha (Thr320) Antibody WB: 1:500, BSA+0.1% Tween20; ON 4ºC+1h RT Cell Signaling Technology Cat# 2581; RRID: AB_330823

Mouse Anti-Rabbit GADPH Monoclonal Antibody, Unconjugated, Clone 6C5 WB: 1:5000, I-Block™; ON 4ºC Millipore Cat# CB1001; RRID: AB_2107426

Phospho-cdc2 (Tyr15) (10A11) Rabbit mAb antibody WB: 1:500, I-Block™; ON 4ºC+1h RT Cell Signaling Technology Cat# 4539; RRID: AB_560953

cdc2 Antibody WB: 1:500, I-Block™; ON 4ºC+1h RT Cell Signaling Technology 1 Cat# 77055; RRID: AB_271633

Cdc25C (F-5) antibody WB: 1:1000, I-Block™; ON 4ºC+1h RT Santa Cruz Biotechnology Cat# sc-55513; RRID: AB_2275797

Anti-PSMC5 antibody produced in rabbit (Called rPSMC5 within this work) WB: 1:500, I-Block™; ON 4ºC+1h RT; IF: 1:250; IHC:1:1000 Sigma-Aldrich Cat# HPA017871; RRID: AB_1857638

Monoclonal ANTI-FLAG® M2 antibody WB: 1:1000, I-Block™; ON 4ºC; IP:0.1µg Sigma-Aldrich Cat# F1804; RRID: AB_262044

Anti-SUMO1 Antibody, clone 21C7 (Called mSUMO1 within this work) WB:1:500, I-Block™; ON 4ºC+1h RT IF:1:100, ON 4ºC Sigma-Aldrich MABS2071

Anti-SUMO2/3 Antibody, clone 8A2 WB:1:500, I-Block™; ON 4ºC+1h RT IF:1:100, ON 4ºC Sigma-Aldrich MABS2039

SUMO-1 (human) polyclonal antibody WB:1:1000, I-Block™; ON 4ºC+1h RT IF:1:700, ON 4ºC Enzo Life Sciences BML-PW0505A-0100

6x-His Tag Monoclonal Antibody (HIS.H8) Invitrogen Catalog # MA1-21315 RRID:AB_557403

Mouse IgG2a Isotype Control from murine myeloma antibody IP: 0.1µg Sigma-Aldrich Cat# M5409; RRID: AB_1163691

Mouse IgG1 Isotype Control from murine myeloma antibody IP: 0.1µg Sigma-Aldrich Cat# M5284; RRID: AB_1163685

Monoclonal Mouse Anti-Thyroid Transcription Factor, Clone 8G7G3/1 antibody IHC: Ready to use Agilent Cat# M3575; RRID: AB_2877699

FLEX Polyclonal Rabbit Anti-Human Thyroglobulin IHC:Ready to use Agilent Cat# IR50961-2

FLEX Monoclonal Mouse anti-human Cytokeratin antibody HC:Ready to use Agilent Cat# IR05361-2; RRID: AB_2868599

FLEX Monoclonal Rabbit Anti-Human Cytokeratin 8/18, Clone EP17/EP30 HC:Ready to use Agilent Cat# IR09461-2

PAX8 Monoclonal Rabbit ,Clone SP348 IHC: 1:100 Gennova Cat# AP10761

Monoclonal Mouse Anti-Human Ki-67 Antigen, Clone MIB-1 antibody IHC: Ready to use Agilent Cat# M7240; RRID: AB_2142367

FLEX Monoclonal Mouse Anti-Human p53 Protein, Clone DO-7 IHC: Ready to use Agilent Cat# IR61661-2

Rabbit Anti-Mouse Immunoglobulins/HRP antibody WB: 1:5000, I-Block™ 1h RT Agilent Cat# P0260; RRID: AB_2636929

Anti-Rabbit IgG (whole molecule)-Peroxidase antibody produced in goat WB: 1:10000, I-Block™ 1h RT Sigma-Aldrich Cat# A0545; RRID: AB_257896

m-IgGκ BP-HRP Antibody WB: 1:1500, I-Block™ 1h RT Santa Cruz Biotechnology Cat# sc-516102; RRID: AB_2687626

b-Tubulin (9F3) Rabbit mAb (Alexa Fluor 647 Conjugate (Called TUBB-647 within this work) 1:300 for 1 hour before last washing and mounting the mitotic cells Cell Signaling Cat# 3624

Recombinant Alexa 647 anti-Sumo1 antibody (Y299) (Called SUMO1-647 within this work) 1:50 ON at 4º Abcam Ab196533

Cy3-AffiniPure Donkey Anti-Rabbit IgG (H+L) antibody IF:1:600 Jackson ImmunoResearch Labs Cat# 711-166-152; RRID: AB_2313568

Alexa Fluor 488 donkey anti-mouse antibody IF:1:400 Jackson ImmunoResearch Labs Cat# 715-546-151; RRID: AB_2340850

Cy3-AffiniPure F(ab')2 Fragment Donkey Anti-Mouse IgG (H+L) (min X Bov,Ck,Gt,GP,Sy Hms,Hrs,Hu,Rb,Rat,Shp Sr Prot) antibody IF: 1:400 Jackson ImmunoResearch Labs Cat# 715-166-151; RRID: AB_2340817

Donkey Anti-Rabbit IgG (H+L) Antibody, Alexa Fluor 488 Conjugated IF: 1:600 Molecular Probes Cat# A-21206; RRID: AB_2535792

Cy3-AffiniPure F(ab')2 Fragment Donkey Anti-Human IgG (H+L) (min X Bov,Ck,Gt,GP,Sy Hms,Hrs,Ms,Rb,Rat,Shp Sr Prot) antibody IF: 1:600 Jackson ImmunoResearch Labs Cat# 709-166-149; RRID: AB_2340538

EnVision™+ Dual Link System-HRP IHC: Ready to use Agilent Cat# K4061

Validation

PIASx (D-12): https://www.scbt.com/es/p/piasx-antibody-d-12

PIAS2b polyclonal antibody: https://www.sigmaaldrich.com/US/en/product/sigma/hpa068792

Aurora A antibody: https://www.abcam.com/products/primary-antibodies/aurora-a-antibody-centrosome-marker-ab1287.html

Phospho-Aurora A (Thr288) (C39D8): https://www.cellsignal.com/products/primary-antibodies/phospho-aurora-a-thr288-c39d8-rabbit-mab/3079

Anti-alpha Tubulin antibody - Microtubule Marker: https://www.abcam.com/products/primary-antibodies/alpha-tubulin-antibody-microtubule-marker-ab18251.html

Monoclonal alpha-Tubulin antibody produced in mouse: https://www.sigmaaldrich.com/ES/es/product/sigma/t5168

Mouse beta-Actin Monoclonal Antibody, Unconjugated, Clone AC-74: https://www.sigmaaldrich.com/ES/en/product/sigma/a5316

Beta3 Tubulin (TU-20) antibody (Called TUBB3 within this work) https://www.scbt.com/p/beta3-tubulin-antibody-tu-20

Gamma Tubulin antibody: https://www.abcam.com/products/primary-antibodies/gamma-tubulin-antibody-ab11317.html

Gamma Tubulin (D-10) antibody: https://www.scbt.com/p/gamma-tubulin-antibody-d-10

Plk1 (F-8) antibody: https://www.scbt.com/p/plk-antibody-f-8

Pericentrin antibody: https://www.abcam.com/products/primary-antibodies/pericentrin-antibody-centrosome-marker-ab4448.html

Recombinant Anti-Bub3 antibody [EPR5319(2)]: https://www.abcam.com/products/primary-antibodies/bub3-antibody-epr53192-ab133699.html

Anti-Centromere Antibody: https://www.antibodiesinc.com/products/anti-centromere-protein-antibody-15-234

Phospho-Histone H3 (Ser10) Antibody: https://www.cellsignal.com/products/primary-antibodies/phospho-histone-h3-ser10-antibody/9701

Anti-Histone H3 Antibody, Unconjugated: https://www.cellsignal.com/products/primary-antibodies/histone-h3-antibody/9715

PP2A-Calpha/beta (0.T.118) antibody: https://www.scbt.com/es/p/pp2a-calpha-beta-antibody-0-t-118

p-PP2A-Calpha/beta (F-8) antibody: https://www.scbt.com/es/p/p-pp2a-calpha-beta-antibody-f-8

PP1 (E-9) antibody: https://www.scbt.com/es/p/pp1-antibody-e-9

Phospho-PP1alpha (Thr320) Antibody: https://www.cellsignal.com/products/primary-antibodies/phospho-pp1a-thr320-antibody/2581

Mouse Anti-Rabbit GADPH Monoclonal Antibody, Unconjugated, Clone 6C5: https://www.merckmillipore.com/ES/es/product/Anti-GAPDH-Mouse-mAb-6C5,EMD_BIO-CB1001

Phospho-cdc2 (Tyr15) (10A11) Rabbit mAb antibody: https://www.cellsignal.com/products/primary-antibodies/phospho-cdc2-tyr15-10a11-rabbit-mab/4539

cdc2 Antibody: https://www.cellsignal.com/products/primary-antibodies/cdc2-antibody/77055

Cdc25C (F-5) antibody: https://www.scbt.com/p/cdc25c-antibody-f-5

Anti-PSMC5 antibody produced in rabbit (Called rPSMC5 within this work): https://www.sigmaaldrich.com/ES/es/product/sigma/hpa017871

Monoclonal ANTI-FLAG® M2 antibody: https://www.sigmaaldrich.com/ES/en/product/sigma/f1804

Anti-SUMO1 Antibody, clone 21C7 (Called mSUMO1 within this work): https://www.sigmaaldrich.com/US/en/product/mm/mabs2071

Anti-SUMO2/3 Antibody, clone 8A2: https://www.sigmaaldrich.com/ES/es/product/mm/mabs2039

SUMO-1 (human) polyclonal antibody: https://www.enzolifesciences.com/BML-PW0505A/sumo-1-human-polyclonal-antibody/

6x-His Tag Monoclonal Antibody (HIS.H8): https://www.thermofisher.com/antibody/product/6x-His-Tag-Antibody-clone-HIS-H8-Monoclonal/MA1-21315

Monoclonal Mouse Anti-Thyroid Transcription Factor, Clone 8G7G3/1 antibody: https://www.agilent.com/en/product/immunohistochemistry/antibodies-controls/primary-antibodies/thyroid-transcription-factor-(autostainer-link-48)-76283

FLEX Polyclonal Rabbit Anti-Human Thyroglobulin: https://www.agilent.com/cs/library/packageinsert/public/115644002.PDF

FLEX Monoclonal Mouse anti-human Cytokeratin antibody HC:Ready to use Agilent Cat# IR05361-2; RRID: AB_2868599

FLEX Monoclonal Rabbit Anti-Human Cytokeratin 8/18, Clone EP17/EP30: https://www.agilent.com/cs/library/packageinsert/public/124420002.PDF

PAX8 Monoclonal Rabbit ,Clone SP348 IHC: 1:100 Gennova Cat# AP10761

Monoclonal Mouse Anti-Human Ki-67 Antigen, Clone MIB-1: https://www.agilent.com/cs/library/packageinsert/public/105303004.PDF

FLEX Monoclonal Mouse Anti-Human p53 Protein, Clone DO-7: https://www.agilent.com/cs/library/packageinsert/public/117348002.PDF

b-Tubulin (9F3) Rabbit mAb (Alexa Fluor 647 Conjugate (Called TUBB-647 within this work): https://www.cellsignal.com/products/antibody-conjugates/b-tubulin-9f3-rabbit-mab-alexa-fluor-647-conjugate/3624

Recombinant Alexa 647 anti-Sumo1 antibody (Y299) (Called SUMO1-647 within this work): https://www.abcam.com/products/primary-antibodies/alexa-fluor-647-sumo-1-antibody-y299-ab196533.html

Eukaryotic cell lines

Policy information about cell lines and Sex and Gender in Research

Cell line source(s)

Cell Line:

Human: MB-1 DSMZ Cat# ACC-638;RRID: CVCL_2109
Human: 8305C ECACC Cat# 94090183;RRID: CVCL_1053
Human: BHT-101 DSMZ Cat# ACC-279;RRID: CVCL_1085
Human: CAL-62 DSMZ Cat# ACC-448;RRID: CVCL_1112
Human: BCPAP DSMZ Cat# ACC-273;RRID: CVCL_0153
Human: FTC-238 ECACC Cat# 94060902;RRID: CVCL_2447
Human: PANC-1 ECACC Cat# 32190102;RRID:CVCL_0480

Primary Cultures: (T-NT, Normal Thyroid; T-MNG, Multinodular Goiter or Thyroid Follicular Nodular Disease; T-PC, Papillary Thyroid Carcinoma, T-M, lymph node Metastasis of PTC; FTC, Follicular Thyroid Carcinoma; T-UC, Anaplastic (Undifferentiated) Thyroid Carcinoma)

T-NT9 (Male)
T-NT16 (Female)
T-NT19 (Female)
T-NT22 (Male)
T-NT23 (Female)
T-NT24 (Female)
T-NT31 (Male)
T-PC18 (Male)
T-PC20 (Female)
T-PC21 (Female)
T-PC25 (Male)
T-PC27 (Female)
T-PC28 (Female)
T-PC29 (Female)
T-PC33 (Male)
T-NT2 (Female)

T-NT36 (Female)
T-NT39 (Female)
T-NT47 (Male)
T-NT52 (Female)
T-NT66 (Male)
T-GD9 (Female)
T-MNG94 (Female)
T-MNG95 (Female)
T-MNG96 (Female)
T-MNG100 (Female)
T-MNG170 (Female)
T-MNG180 (Female)
T-MNG182 (Female)
T-PC46 (Female)
T-PC48 (Female)
T-PC64 (Female)
T-PC65 (Female)
T-PC95 (Female)
T-PC115 (Female)
T-PC123 (Male)
T-M19 (Female)
T-FC7 (Female)
T-UC1 (Female)
T-UC2 (Female)
T-UC3 (Female)
T-UC7 (Female)
T-UC8 (Female)

Authentication	Cell lines used were authenticated by STR and mutational profile analysis.
Mycoplasma contamination	Cell lines tested negative for mycoplasma
Commonly misidentified lines (See ICLAC register)	No commonly misidentified lines were used

Animals and other research organisms

Policy information about studies involving animals; ARRIVE guidelines recommended for reporting animal research, and Sex and Gender in Research

Laboratory animals	Nine 7-week-old NOD-SCID female mice (NOD.CB-17-PrkdcSCID / Rj) were obtained from Janvier Labs. Twenty 6-week-old female NSG mice (NOD.Cg-PrkdcSCID Il2rgtm1Wjl/SzJ) were obtained from Charles River. Mice were housed with an artificial 12 hr light/12 hr dark cycle, under controlled temperature (22–24 °C) and humidity conditions (40%) and allowed to free access to standard laboratory chow and tap water. When 8-week-age, mice were orthotopically implanted.
Wild animals	No wild animals were used.
Reporting on sex	Female
Field-collected samples	No field-collected samples were used.
Ethics oversight	Animal studies were reviewed and approved under the Procedures Act nº 15003/14/005 (to CVA), granted by Galicia Regional Government, following NC3Rs' ARRIVE guidelines. Our orthotopic ATC Patient-derived Xenograft model has a time window defined by the approvals of the Ethical Committee. The Endpoint was 9-10 weeks from the orthotopic implantation of the tumor, with daily observation starting at week 9 checking for endpoints such as >20% weight loss or being moribund. The primary outcome was tumor growth/volume, which was monitored in the IVIS. A secondary purpose was established for each PCT in order to reduce the number of mice used, following the 3R rule:

Note that full information on the approval of the study protocol must also be provided in the manuscript.

Clinical data

Policy information about clinical studies
All manuscripts should comply with the ICMJEguidelines for publication of clinical research and a completedCONSORT checklist must be included with all submissions.

Clinical trial registration	N/A
Study protocol	N/A

Data collection	N/A
Outcomes	N/A

Plants

Seed stocks	N/A
Novel plant genotypes	N/A
Authentication	N/A

Flow Cytometry

Plots

Confirm that:

- [x] The axis labels state the marker and fluorochrome used (e.g. CD4-FITC).
- [x] The axis scales are clearly visible. Include numbers along axes only for bottom left plot of group (a 'group' is an analysis of identical markers).
- [x] All plots are contour plots with outliers or pseudocolor plots.
- [x] A numerical value for number of cells or percentage (with statistics) is provided.

Methodology

Sample preparation	As described in Methods, a minimum of 240,000 cells were seeded per condition or sample; the procedure was adapted from (Zhao et al., 2007). Cells were stained with 1 mL DAPI solution (1 µg/mL DAPI [D9542, Sigma-Aldrich] and 0.1% Triton (T8787, Sigma-Aldrich) in PBS) for every 1106 cells. A total volume of 300 µL of each sample was distributed into a 96-well multiplate and analyzed.
Instrument	BD Accuri™ C6 cytometer (BD Biosciences) with violet laser (405 nm)
Software	Data were analyzed in FlowJo software v10
Cell population abundance	N/A
Gating strategy	1.) SSC vs. FSC gating to exclude debris. 2.) FSC-H vs. FSC-A gating to exclude doublets.<br>3.) FSC-H vs. DAPI gating to analyse cell cycle

- [x] Tick this box to confirm that a figure exemplifying the gating strategy is provided in the Supplementary Information.
