# Supplementary material for: dsRNAi-mediated silencing of PIAS2beta specifically kills anaplastic carcinomas by mitotic catastrophe
Source: Nat Commun. 2024 May 14;15:3736. doi: 10.1038/s41467-024-47751-1 (PMC11094195; doi:10.1038/s41467-024-47751-1)
Supplement: Supplementary file 3 — Description of Additional Supplementary Files [file 41467_2024_47751_MOESM3_ESM.pdf]

Supplementary Data 1. 1.1 Identified proteins in 2D electrophoresis followed by Mass spec. 1.2 *PIAS2* mRNA isoforms from Ensembl and NCBI. Right: Isoforms recognized by *PIAS2b*-dsRNAi and T-*PIAS2b*-shRNA, and by each subset of RT-qPCR primers (*PIAS2b*, *PIAS2a*, *global PIAS2*). 1.3 BLAST results for each dsRNAi sequence (*PIAS2b*-dsRNAi 1 and 2; ns-dsRNAi).

Supplementary Data 2. 2.1 Primary cultures, tissue surplus, and cell lines: clinical, genetic background and mutational characteristics of patients' and tissues of origin. Cell lines: name genotype, SSTRs for identification, and efficiency of transfection. 2.2 Analysis of the Thyroid and Non-Thyroid Cell Lines used in the manuscript, with bioinformatic data retrieved from from original dataset in Cohen-Sharir, Y., McFarland, J.M., Abdusamad, M. et al. Nature, 2021: Aneuploidy classification and Aneuploidy score; Ploidy.

Supplementary Data 3. LC-MS/MS Triple TOF. Proteomic assay 1 (PA1) and 2 (PA2). PA1 (n =5 independent samples per condition). 3.1 Proteins associated to PIAS2 at DT/R-6h after immunoprecipitation; quantification performed by M1 Spectrum Count (Fisher's Exact Test from Scaffold). 3.2 From 3.1, proteins with QUANTscore >1.5 PIAS2 binding in Spectrum Count_PA1 presenting a High score SUMO Consesus site in GPS-Sumo Prediction GPS 2.0 Online. 3.3 PA1 quantified by SWATH (T-test from Marker's View). 3.4 From 3.3, proteins with adjPvalue<0.11 for PIAS2 binding in SWATH_PA1 presenting a High score SUMO Consesus site in GPS-Sumo Prediction GPS 2.0 Online Service. 3.5 Proteomic assay 2 (PA2) (n = 3 independent samples per condition): EGFP-PIAS2b pull-downs after GFP-Trap, quantification performed by M1 Spectrum Count (Fisher's Exact Test from Scaffold). 3.6 From 3.5, proteins with QUANTscore >1.5 in EGFP-PIAS2b Spectrum Count_PA2 presenting a High score SUMO Consesus site in GPS-Sumo Prediction GPS 2.0 Online Service. 3.7□3.9 Progressive sheets show data-cross analysis of both quantifications of Proteomic Assay 1, of Proteomic Assay 1+2, and the enriched Reactome and STRING pathways.

Supplementary Data 4. Proteomic Assay 3 (n = 3 independent samples per condition): Mitotic (DT/R- 5h) proteins identified as SUMOylated (retained) or NOT SUMOylated (flow-through) in ns-dsRNAi and *PIAS2b*-dsRNAi. 4.1 Quantification performed by M1 Spectrum Count (Fisher's Exact Test from Scaffold). 4.2 □ 4.7 Progressive sheets show Enhanced SUMOylated proteins in 1 compared to 2 (Score >1.5), only if were detected in (2 or 4); Reduced SUMOylated proteins in 2 compared to 1 (Score <0.5), only if were detected in either (1 or 3). Data-cross analysis of selected proteins from Proteomic Assay 1+2+3, and the enriched Reactome and STRING pathways.

Supplementary Movie 1. Time-lapse video recording of two thyroid cancer cell lines stained with SirDNA, starting at day 2 after transfection of *ns*-dsRNAi or *PIAS2b*-dsRNAi: Video S1.1: The ATC cell line CAL-62 (mitotic catastrophe); Video S1.2: The PDTC (papillary origin) cell line B-CPAP (no effect).

Supplementary Movie 2. Time-lapse video recording of two ATC primary cultures starting at day 2 after transfection of ns-dsRNAi or *PIAS2b*-dsRNAi. Video S2.1: The T-UC2 primary culture, starting at day 2 after co-transfection of Histone H2B-GFP (green fluorescence) and ns-dsRNAi or *PIAS2b*-dsRNAi. Video S2.2: The T-UC1 primary culture treated with ns-dsRNAi or *PIAS2b*-dsRNAi and stained with SirDNA. Video S2.3: The T-UC2 primary culture treated with ns-dsRNAi or *PIAS2b*-dsRNAi and stained with SirDNA.

Supplementary Movie 3. Image 3D surface rendering of selected mitotic cells obtained after staining, confocal microscopy, deconvolution and analysis at Imaris.
